# CT imaging-derived phenotypes for abdominal muscle and their association with age and sex in a medical biobank

**DOI:** 10.1038/s41598-024-64603-6

**Published:** 2024-06-26

**Authors:** Phuong T. Vu, Chantal Chahine, Neil Chatterjee, Matthew T. MacLean, Sophia Swago, Abhi Bhattaru, Elizabeth W. Thompson, Anooshey Ikhlas, Edith Oteng, Lauren Davidson, Richard Tran, Mohamad Hazim, Pavan Raghupathy, Anurag Verma, Jeffrey Duda, James Gee, Valerie Luks, Victoria Gershuni, Gary Wu, Daniel Rader, Hersh Sagreiya, Walter R. Witschey, The Penn Medicine Biobank, Daniel J. Rader, Marylyn D. Ritchie, JoEllen Weaver, Nawar Naseer, Afiya Poindexter, Khadijah Hu-Sain, Yi-An Ko, JoEllen Weaver, Meghan Livingstone, Fred Vadivieso, Stephanie DerOhannessian, Teo Tran, Julia Stephanowski, Monica Zielinski, Ned Haubein, Joseph Dunn, Anurag Verma, Colleen Morse Kripke, Marjorie Risman, Renae Judy, Anurag Verma, Shefali S. Verma, Yuki Bradford, Scott Dudek, Theodore Drivas

**Affiliations:** 1 Department of Radiology, Perelman School of Medicine, Perelman Center for Advanced Medicine, University of Pennsylvania, 3400 Civic Center Boulevard, Philadelphia, PA 19104 USA; 2 Department of Genetics, Perelman School of Medicine, University of Pennsylvania, Philadelphia, PA USA; 3 Department of Medicine, Perelman School of Medicine, University of Pennsylvania, Philadelphia, PA USA; 4 Perelman School of Medicine, University of Pennsylvania, Philadelphia, PA USA; 5 Wallace H. Coulter Department of Biomedical Engineering, Georgia Institute of Technology and Emory University, Atlanta, GA USA

**Keywords:** Biomedical engineering, Computational biology and bioinformatics, Biomarkers, Medical research

## Abstract

The study of muscle mass as an imaging-derived phenotype (IDP) may yield new insights into determining the normal and pathologic variations in muscle mass in the population. This can be done by determining 3D abdominal muscle mass from 12 distinct abdominal muscle regions and groups using computed tomography (CT) in a racially diverse medical biobank. To develop a fully automatic technique for assessment of CT abdominal muscle IDPs and preliminarily determine abdominal muscle IDP variations with age and sex in a clinically and racially diverse medical biobank. This retrospective study was conducted using the Penn Medicine BioBank (PMBB), a research protocol that recruits adult participants during outpatient visits at hospitals in the Penn Medicine network. We developed a deep residual U-Net (ResUNet) to segment 12 abdominal muscle groups including the left and right psoas, quadratus lumborum, erector spinae, gluteus medius, rectus abdominis, and lateral abdominals. 110 CT studies were randomly selected for training, validation, and testing. 44 of the 110 CT studies were selected to enrich the dataset with representative cases of intra-abdominal and abdominal wall pathology. The studies were divided into non-overlapping training, validation and testing sets. Model performance was evaluated using the Sørensen–Dice coefficient. Volumes of individual muscle groups were plotted to distribution curves. To investigate associations between muscle IDPs, age, and sex, deep learning model segmentations were performed on a larger abdominal CT dataset from PMBB consisting of 295 studies. Multivariable models were used to determine relationships between muscle mass, age and sex. The model's performance (Dice scores) on the test data was the following: psoas: 0.85 ± 0.12, quadratus lumborum: 0.72 ± 0.14, erector spinae: 0.92 ± 0.07, gluteus medius: 0.90 ± 0.08, rectus abdominis: 0.85 ± 0.08, lateral abdominals: 0.85 ± 0.09. The average Dice score across all muscle groups was 0.86 ± 0.11. Average total muscle mass for females was 2041 ± 560.7 g with a high of 2256 ± 560.1 g (41–50 year old cohort) and a change of − 0.96 g/year, declining to an average mass of 1579 ± 408.8 g (81–100 year old cohort). Average total muscle mass for males was 3086 ± 769.1 g with a high of 3385 ± 819.3 g (51–60 year old cohort) and a change of − 1.73 g/year, declining to an average mass of 2629 ± 536.7 g (81–100 year old cohort). Quadratus lumborum was most highly correlated with age for both sexes (correlation coefficient of − 0.5). Gluteus medius mass in females was positively correlated with age with a coefficient of 0.22. These preliminary findings show that our CNN can automate detailed abdominal muscle volume measurement. Unlike prior efforts, this technique provides 3D muscle segmentations of individual muscles. This technique will dramatically impact sarcopenia diagnosis and research, elucidating its clinical and public health implications. Our results suggest a peak age range for muscle mass and an expected rate of decline, both of which vary between genders. Future goals are to investigate genetic variants for sarcopenia and malnutrition, while describing genotype–phenotype associations of muscle mass in healthy humans using imaging-derived phenotypes. It is feasible to obtain 3D abdominal muscle IDPs with high accuracy from patients in a medical biobank using fully automated machine learning methods. Abdominal muscle IDPs showed significant variations in lean mass by age and sex. In the future, this tool can be leveraged to perform a genome-wide association study across the medical biobank and determine genetic variants associated with early or accelerated muscle wasting.

## Introduction

Sarcopenia is a prevalent but challenging^1^ diagnosis defined by the age-related decline in lean body mass. When the term sarcopenia was first coined in 1989, it was said that “there may be no single feature of age-related decline more striking than the decline in lean body mass in affecting ambulation, mobility, energy intake, overall nutrient intake, and status, independence and breathing”^2^.

Sarcopenia has since increasingly become recognized for its central role in different disease processes, such as cancer and post-operative outcomes, and for its profound effect on quality of life^3,4^. A common quantitative biomarker of the evolving definition of sarcopenia has been muscle loss^5^. There are multimodal tools and techniques for obtaining surrogate measures of muscle mass, relying on different chemical and physical aspects of muscle in different areas of the body^6–11^. However, in vivo measurement and granular phenotyping of abdominal whole-muscle mass at the muscle group-level would fill the persistent need for an objective reference standard for quantitative muscle assessment^12,13^.

Muscle mass quantification from large-scale imaging studies can yield new insights into the normal and pathologic variations in muscle mass, loss of lean muscle mass with age, and biological variation by sex. Muscle imaging derived phenotypes (IDPs) for total muscle and group-level mass may be determined automatically from non-invasive CT and MR imaging studies. Given the increasing volume of medical imaging studies, these could be used opportunistically to screen patients for muscle disease, determine risk, and uncover genetic and environmental factors associated with muscle loss without conferring additional risk of radiation to the patient^13,14^.

Recent studies have quantified abdominal skeletal muscle mass using computed tomography (CT) scans using deep learning^15–18^. In addition to total abdominal muscle mass, semantic (multiclass) segmentations have been developed for imaging analysis and could be used to determine the spatial distribution of individual muscle groups, yet the 3D distributions of group-level abdominal muscle mass IDPs have not been reported. Additionally, recent deep learning advances permit the classification of CT studies for fully automated analysis, determining whether individual scans were performed with contrast enhancement^13^, and using anatomical landmarks to provide standardized reporting of IDPs for abdominal muscle groups that may lie only partly in the imaging field-of-view. These techniques should be integrated with muscle segmentation algorithms to fully automate analysis. Finally, the 3D distribution of individual abdominal muscle groups by age and sex has not been determined.

The purpose of this study was to develop a deep learning algorithm to determine the 3D distribution of 12 abdominal muscle groups on abdominal CT. Several residual network architectures for semantic segmentation of muscle mass were analyzed for performance in comparison to the ground truth data set. In a set of 295 patients from a medical biobank, we determined the relationships between individual muscle group-level IDPs, age and sex.

## Methods

### Patient imaging data in the Penn Medicine Biobank (PMBB)

The PMBB is a research study that recruits participants throughout the Penn Medicine Health System (Philadelphia, PA) by enrolling them at the time of outpatient visits^45^. Patients complete a questionnaire, donate a blood sample, allow researchers access to their electronic health record, and agree to future recontact. The PMBB is a racially diverse cohort with black patients comprising nearly 25% of participants. This study was approved by the Institutional Review Board of the University of Pennsylvania and all patients have given informed consent to participate in this study. All methods were performed in accordance with the relevant guidelines and regulations.

### Summary of imaging data

The ground truth data set consisted of 110 studies with Current Procedural Terminology (CPT) codes 74,176 (CT abdomen and pelvis without contrast, n = 32), 74,177 (CT abdomen and pelvis with contrast, n = 73), and 74,178 (CT abdomen and pelvis with and without contrast, n = 5). Within each study, series were excluded if they were not in the axial orientation, used high-pass reconstruction kernels, or were axial series with a slice thickness of less than 2 mm (46% of all series were excluded based on these criteria). This procedure excluded studies with too much noise. De-identification of the imaging data was performed with the software dcm2niix^19^. The range of attenuation used for skeletal muscle was from − 29 to 150 Hounsfield units^48^. Trainees (A.I., E.O., L.D., R.T., M.H., P.R.) labeled all studies, encompassing 2057 axial CT slices and 980 labels. To determine inter-rater variability, 6 studies were labeled by all trainees, and the resulting segmentations were evaluated for the repeatability of the ground truth (Supplementary Figs. S2 and S3, Supplementary Information). All labels were reviewed for additional corrections by an imaging scientist (W.R.W.) and a subset were reviewed by a board-certified radiologist (H.S.).

Of the 110 studies, 44 were enrichment studies showing intra-abdominal pathologies, some of which affect abdominal muscle anatomy and others of which demonstrate common abdominal pathologies. Pathologies were selected by querying radiology reports among PMBB participants for confirmed diagnoses. The enrichment dataset included diverticulitis, sarcopenia, colon cancer, umbilical hernia, cholelithiasis, cholecystitis, ulcerative colitis, intestinal fistula, abdominal abscess, small bowel obstruction, pyelonephritis, Crohn’s disease, diverticulitis with hemorrhage, hepatomegaly, splenomegaly, short bowel syndrome, kidney stone, ventral hernia, and perianal fistula (Supplementary Fig. S1).

To investigate associations between muscle IDPs, age, and sex, segmentations by the deep learning model were performed on a larger abdominal CT dataset (analysis set) from PMBB consisting of 303 studies (90 of these were also part of the training, validation, and test dataset). The new analysis set consisted of studies with CPT codes^20^ 74,176 (CT abdomen and pelvis) (n = 57), 74,177 (CT abdomen and pelvis with contrast) (n = 217), 74,178 (CT abdomen and pelvis without contrast in one or both body regions, followed by contrast material(s) and further sections in one or both body regions) (n = 14), 74,150 (CT abdomen and pelvis without dye) (n = 4), 74,170 (CT abdomen and pelvis without and with dye) (n = 3). The automated segmentations were reviewed by a research track radiology resident (N.C) and poor segmentation results were removed. 8 scans were excluded prior to statistical analysis due to false positives identified by the model, so the finalized analysis set consisted of 295 studies.

### Deep learning architectures and training

Segmentations were performed using 3D Slicer (v. 4.10.0)^21^ and confirmed using ITK-SNAP (v. 3.8.0)^22^. Data preprocessing steps included adjusting the window level (window: 400, level: 40), enforcing consistent voxel ordering, and extracting 2D axial slices from the original NIfTI volumes. Overall, the training set had 76 studies (828 slices), the validation set had 19 studies (207 slices), the test set had 15 studies (162 slices), and the analysis set had 295 studies. Five-fold cross validation was performed on the best-performing model using the training and validation studies (n = 95, 1035 slices). 5 repeats of fivefold cross-validation were performed, with results reported as the average Dice score across the 5 validation folds.

**Figure 1:**
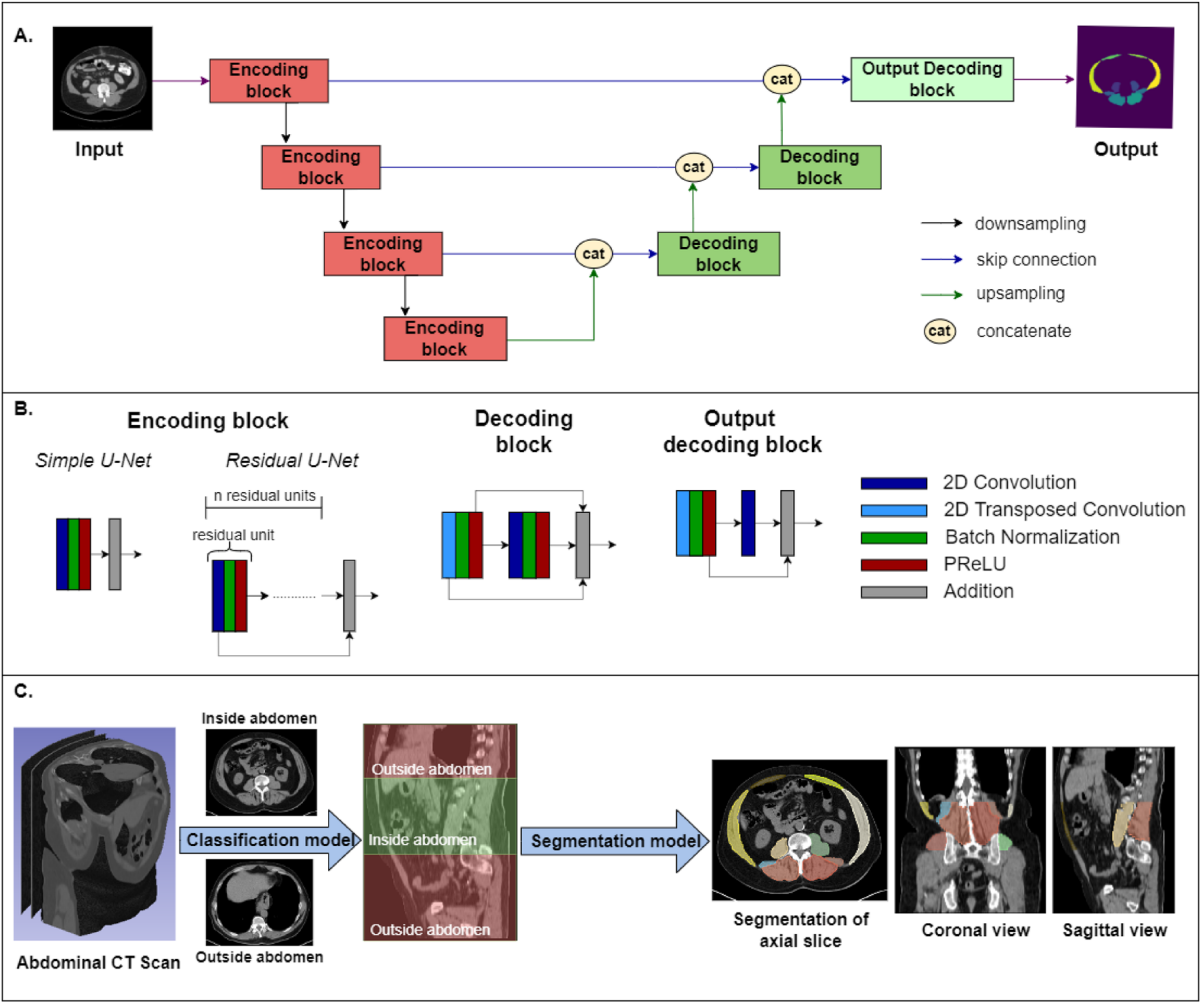
Fully automatic determination of abdominal muscle IDPs from a medical biobank. (**A**) Architecture of the machine learning algorithm for multiclass semantic segmentation of abdominal muscles. There are 4 encoding blocks in the descending portion of the cascade and 3 decoding blocks in the upward portion. Encoding and decoding blocks were connected at each resolution layer. (**B**). Details of the encoding and decoding blocks. The encoding block of the simple U-Net consists of 2D convolutional layers, batch normalization, activation (PReLU, Parametric Rectified Linear Unit), and addition. The encoding block of the Residual U-Net had n residual units where n = 1, 2, 3, 4 and each residual unit consists of the same layers as the simple U-Net encoding block, but the first layer is connected to the last addition layer. The decoding block was concatenated with the encoding block output at each level via skip connections and subsequently underwent batch normalization, activation, convolution and addition. (**C**) The fully automatic approach reviews all series of an abdominal CT study, determines whether each series was unenhanced, and subsequently identifies the abdominal cavity between the lung and the inferior aspect of the L5 disc. For unenhanced series, segmentation is performed in the abdominal cavity as described in (**A**) and (**B**).

Multiclass semantic segmentation was performed for 12 groups of muscles from 2D axial slices. Five models adapted from the U-Net architecture were developed and validated using the PyTorch-based framework MONAI (version 0.9.0): Simple U-Net and Residual U-Nets with 1 to 4 residual units^23,24^. The building blocks of these models are shown in Fig. 1A,B. The descending portion consists of 4 encoding blocks and the ascending portion consists of 3 decoding blocks. For the simple U-Net, the encoding block is composed of 2D convolutional layers, batch normalization, activation (PReLU, Parametric Rectified Linear Unit), and addition. For the Residual U-Nets, the encoding block has n residual units where n = 1, 2, 3, 4. Each residual unit had the same layers as the simple U-Net encoding block with the addition of the connection between the first layer and the last addition layer. The Decoding block was concatenated with the encoding block output at each level via skip connections and subsequently underwent batch normalization, activation, convolution and addition.

For each voxel, the networks output the probabilities that it belongs to one of the muscle groups or the foreground. The function argmax in PyTorch^25^ (version 1.12.0) was used to obtain the label that gave the maximum probability value. Label hole-filling was performed using the Binary Fillhole Image Filter in SimpleITK (version 21.2.4)^26–28^. All segmentation models were initially trained using the Adam optimizer using a learning rate of 10^–4^ for 100 epochs. Training was performed from scratch with 128 validation steps per epoch. Performance at the end of the 100th epoch was compared to determine the best performing model.

After comparing model performance, the best-performing model was trained for another 200 epochs with the first half at the learning rate of 10^–5^ and the second half at the learning rate of 10^–6^. Data augmentation strategies included random transformations zoom = [− 0.2, 0.2] and rotation = [− 20°, 20°] and were applied with a probability of 80%. The final performance was evaluated by computing the Dice scores between the model output and ground truth on the 15 studies in the test set, which consists of 162 axial slices.

### Segmentation of the analysis dataset

Muscle IDPs were determined in the analysis dataset by combining a previously developed classification method developed by MacLean et al.^29^ to delineate the abdominal cavity with the segmentation model as shown in Fig. 1. The abdominal cavity was defined as the region between the inferior aspect of the lung and inferior aspect of the L5 vertebrae. 3D abdominal muscle group segmentation was then done between these two planes, producing distinct labels for the left and right psoas, quadratus lumborum, erector spinae, gluteus medius, rectus abdominis, and lateral abdominals. The abdominal cavity^29^ was delineated automatically using machine learning as previously reported in the context of abdominal fat. New methods were developed for segmentation of the abdominal muscles as described under *Deep Learning Architectures and Training*. Muscle volume was automatically determined from the labeled images and muscle mass was calculated by multiplying the measured muscle volume by a muscle density of 1.06 kg/L^46^.

### Statistical analysis

Linear regression analysis was used to determine the association between manual and automatically determined cross-sectional areas, and intraclass correlation coefficients (ICC) were measured. Statistical significance was determined at *P* < 0.05. All statistical analyses were performed using R (R Core Team, version 4.1.2; Foundation for Statistical Computing, Vienna, Austria).

Descriptive statistics were determined for muscle mass by age (grouped by decile) and sex. Associations between muscle mass, age, and sex were determined by using Pearson correlation coefficients. To account for the correlation between muscle mass and height/BMI, height-adjusted muscle size was obtained by dividing muscle mass by height squared, and BMI-adjusted muscle size was obtained by dividing muscle mass by BMI. The Pearson correlation coefficients between adjusted muscle mass and age were then obtained and compared with the absolute muscle mass case. We investigated the association between height-adjusted skeletal muscle mass, BMI and sex, $SMM={\beta }_{0}+{\beta }_{1} \times BMI+{\beta }_{2} \times sex+{\beta }_{3} \times sex \times BMI$^47^.

## Results

### Cohort characteristics

At the time of imaging, the average patient age was 59.3 ± 14.1 years with an age range of 18–94 in the training dataset (n = 110) and 59.1 ± 14.4 years with a range of 18–95 in the analysis dataset (n = 295). There were 71 females (57.3%) and 53 males (42.8%) in the training dataset and 162 females (54.9%) and 133 males (45.1%) in the analysis set. As described in methods, the training set was used to train the deep learning algorithm and the analysis set was used to determine associations between abdominal muscle and age and sex.

### Performance of residual U-Net

**Figure 2:**
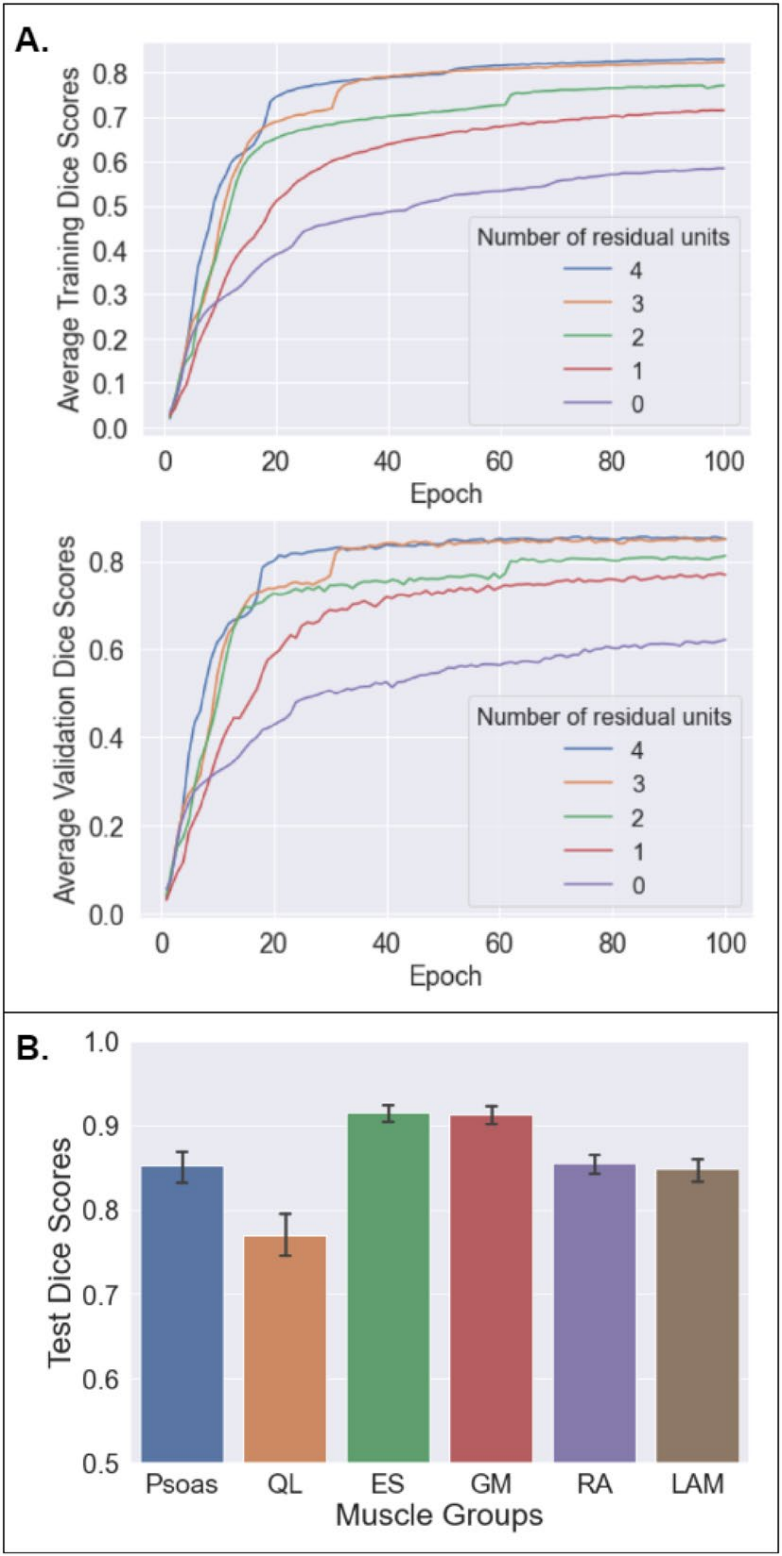
Performance of the residual network shown in Fig. 1A for multiclass semantic segmentation of abdominal muscle groups. (**A**) The average training and validation Dice score during training with 0–4 residual units. Additional residual units improve performance but with diminishing returns. (**B**) Final Dice scores in the testing set (n = 162 slices in 15 studies).

**Figure 3:**
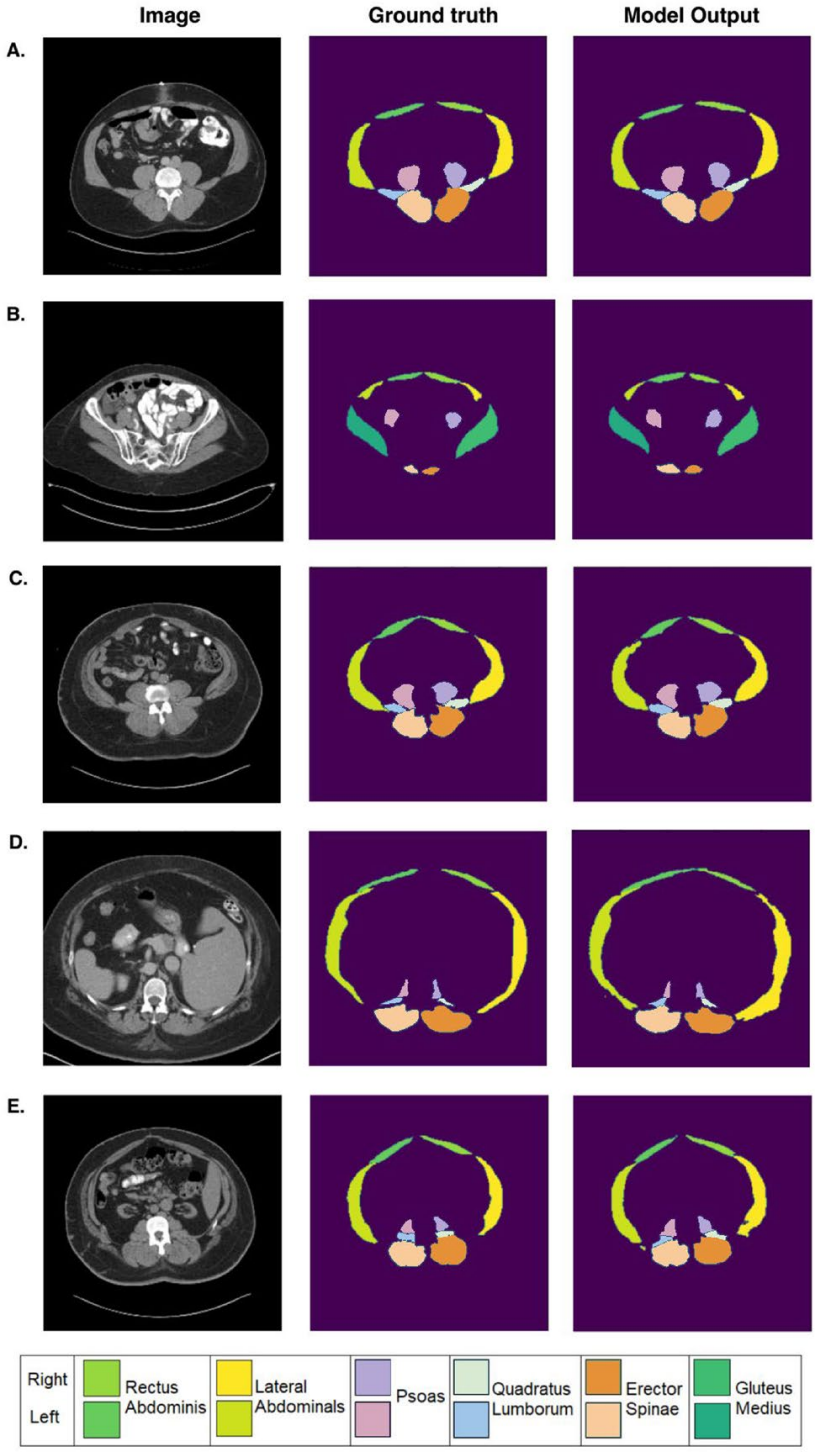
Examples of group-level abdominal muscle segmentation on axial abdominal CT scans in 5 representative patients. The first column shows axial abdominal CT images at different levels in patients. The second column shows ground truth labels, and the third column shows segmentations performed using machine learning. The corresponding Dice scores for each case are as follows: (**A**) Psoas: 0.969 (Left), 0.963 (Right); Quadratus Lumborum (QL): 0.922 (Left), 0.946 (Right); Erector Spinae (ES): 0.950 (Left), 0.946 (Right); Gluteus Medius (GM): N/A; Rectus abdominis (RA): 0.853 (Left), 0.868 (Right); Lateral abdominals (LA): 0.960 (Left), 0.947 (Right). (**B**) Psoas: 0.883 (Left), 0.864 (Right); QL: N/A; ES: 0.842 (Left), 0.782 (Right); GM: 0.912 (Left), 0.910 (Right); RA: 0.904 (Left), 0.898 (Right); LA: 0.740 (Left), 0.764 (Right). (**C**) Psoas: 0.915 (Left), 0.901 (Right); QL: 0.942 (Left), 0.912 (Right); ES: 0.968 (Left), 0.935 (Right); GM: N/A; RA: 0.890 (Left), 0.906 (Right); LA: 0.893 (Left), 0.916 (Right). (**D**) Psoas: 0.922 (Left), 0.904 (Right); QL: 0.887 (Left), 0.847 (Right), ES: 0.964 (Left), 0.952 (Right); GM: N/A; RA: 0.894 (Left), 0.907 (Right); LA: 0.911 (Left), 0.876 (Right). (**E**) Psoas: 0.871 (Left), 0.742 (Right); QL: 0.881 (Left), 0.772 (Right); ES: 0.930 (Left), 0.941 (Right); GM: N/A; RA: 0.834 (Left), 0.896 (Right); LA: 0.848 (Left), 0.886 (Right).

For U-Net models with 0, 1, 2, 3, 4 residual units, the average training Dice scores were (0) 0.584, (1) 0.715, (2) 0.772, (3) 0.824, (4) 0.831, and validation Dice scores were (1) 0.583, (2) 0.715, (3) 0.771, (4) 0.823, (5) 0.830 respectively. The Dice scores for the highest-performing model (U-Net with 4 residual units) in the test set were highest for the erector spinae (0.916 ± 0.071), followed by the gluteus medius (0.904 ± 0.075), the rectus abdominis (0.855 ± 0.075), the psoas (0.852 ± 0.127), the lateral abdominals (0.849 ± 0.088), and the quadratus lumborum (0.770 ± 0.138) (Fig. 2). The average Dice score across all muscle groups in the test set was 0.857 ± 0.107. The Dice scores obtained from five-fold cross validation (calculated across the average Dice scores from each validation fold) were 0.918 ± 0.004 for the erector spinae, 0.897 ± 0.051 for the gluteus medius, 0.856 ± 0.011 for the lateral abdominal muscle region, 0.848 ± 0.020 for the psoas, 0.835 ± 0.026 for the rectus abdominis, and 0.749 ± 0.017 for the quadratus lumborum. The average Dice score across all muscle groups after cross validation was 0.851 ± 0.013. The Dice scores for the left and right side of each muscle group is shown in Table 1. Representative segmentation results are shown in Fig. 3.

**Table 1:** Dice scores for the highest-performing residual U-Net model—4 residual units (results are mean ± standard deviation)

	Psoas		Quadratus lumborum		Erector spinae		Gluteus medius		Rectus abdominis		Lateral abdominals	
	L	R	L	R	L	R	L	R	L	R	L	R
Test set (n = 162 slices from 15 studies)	0.868 ± 0.110	0.837 ± 0.140	0.785 ± 0.131	0.763 ± 00.146	0.913 ± 0.088	0.918 ± 0.049	0.912 ± 0.050	0.896 ± 0.094	0.854 ± 0.081	0.856 ± 0.070	0.855 ± 0.075	0.842 ± 0.010
Five-fold cross validation	0.864 ± 0.012	0.832 ± 0.011	0.757 ± 0.010	0.742 ± 0.019	0.918 ± 0.004	0.918 ± 0.005	0.914 ± 0.038	0.882 ± 0.062	0.834 ± 0.033	0.836 ± 0.022	0.857 ± 0.015	0.855 ± 0.007

Means and standard deviations are computed across 2D slices for the reported test set results and across the average Dice scores of five folds for the reported five-fold cross validation results.

**Figure 4:**
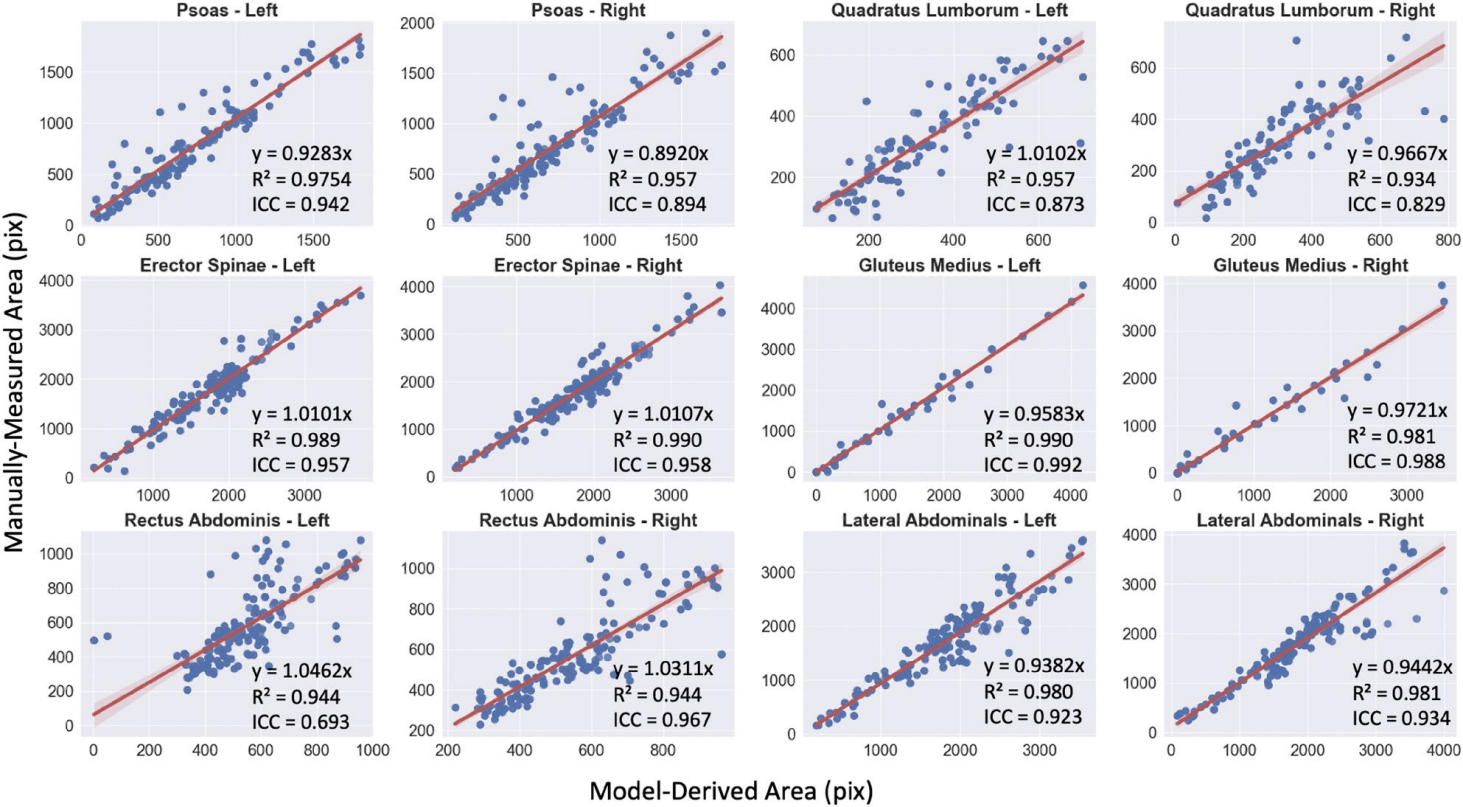
Scatterplots for each abdominal muscle group area showing agreement between ground truth and AI-based segmentations in the test set using optimal parameters (residual network #4, c.f. Fig. 2).

Agreement between the deep learning and manual segmentations is shown in Fig. 4. The intraclass correlation coefficients (ICC) between the number of pixels in the segmented muscle group for deep learning and the manually measured areas are 0.948 for the left psoas and 0.898 for the right psoas, 0.915 for the left and 0.859 for the right quadratus lumborum, 0.957 for the left and 0.958 for the right erector spinae, 0.993 for the left and 0.987 for the right gluteus medius, 0.693 for the left and 0.834 for the right rectus abdominis, and 0.923 for the left and 0.934 for the right lateral abdominal (Table 2). The ICC between model-derived and manually measured total cross-sectional area is 0.9703. The p-values obtained for the total area and all muscle groups are less than 0.0001.

**Table 2:** Intraclass correlation coefficients (ICC) between model-derived and manually measured areas

	Psoas		Quadratus lumborum		Erector spinae		Gluteus medius		Rectus abdominis		Lateral abdominals	
	L	R	L	R	L	R	L	R	L	R	L	R
ICC value	0.942	0.894	0.873	0.829	0.957	0.958	0.992	0.988	0.693	0.834	0.923	0.934

All p values are less than 0.0001.

### Muscle mass distribution by sex

**Figure 5:**
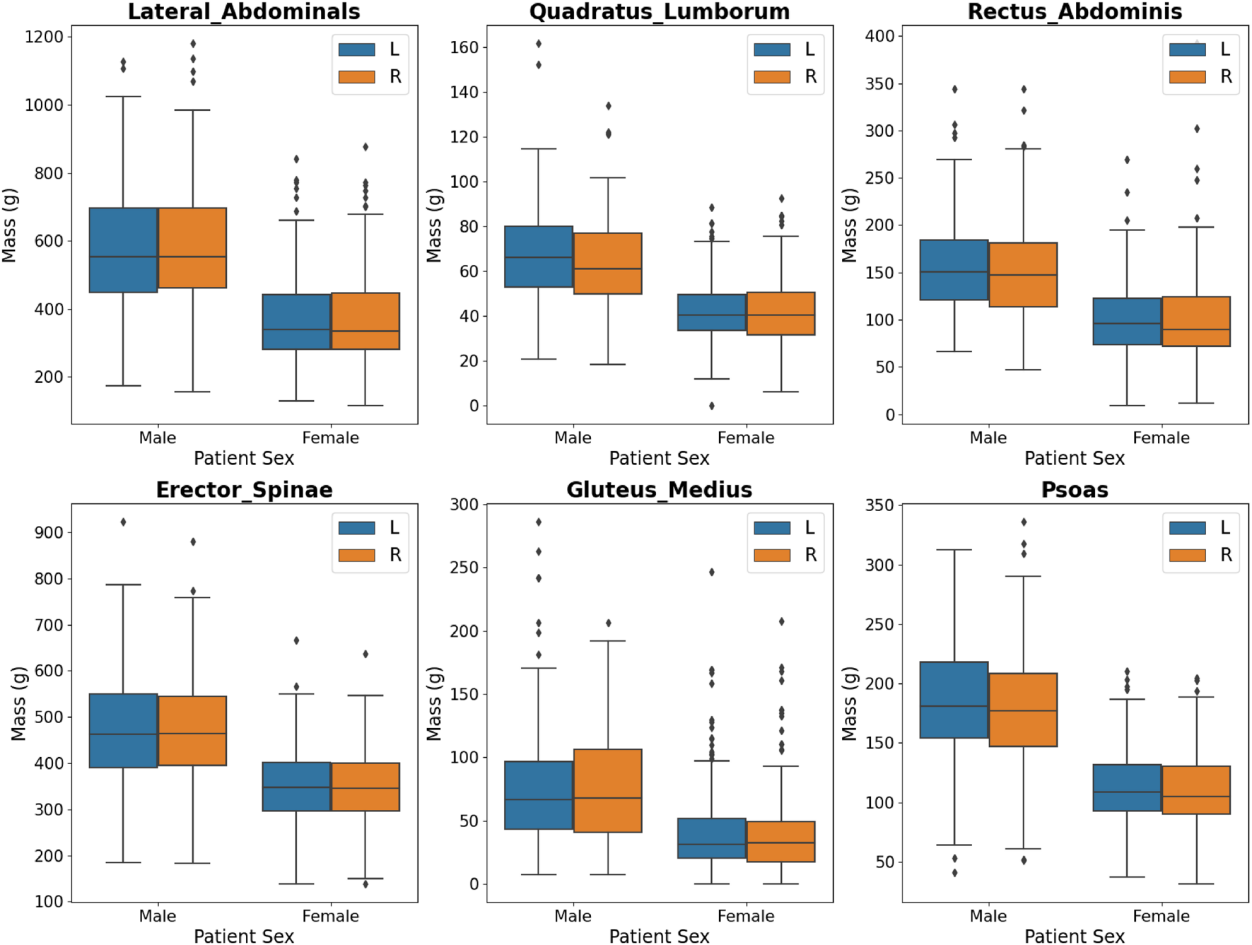
Muscle volume for each muscle group, by hemisphere left (L) or right (R) and sex (male or female), in the training data sets.

As shown in Fig. 5 and Table 3, the muscle groups in order of decreasing muscle mass (mean ± standard deviation in grams) for males are lateral abdominals (1171 ± 350.3), erector spinae (954 ± 242.3), psoas (367 ± 98.8), rectus abdominis (309 ± 99.2), gluteus medius (154 ± 92.9), quadratus lumborum (131 ± 41.7). For females, the muscle groups in order of decreasing mass are lateral abdominals (745 ± 263.1), erector spinae (704 ± 171.0), psoas (224 ± 62.0), rectus abdominis (205 ± 82.3), quadratus lumborum (84.0 ± 27.0), and gluteus medius (82.0 ± 68.7).

**Table 3:** Abdominal muscle mass in grams by age and sex.

	N	Total mass	Erector spinae	Gluteus medius	Lateral abdominals	Psoas	Quadratus lumborum	Rectus abdominis
Sex								
Female	190	2041 ± 560.7	704 ± 171.0	82 ± 68.7	745 ± 263.1	224 ± 62.0	84 ± 27.9	205 ± 82.3
Male	156	3086 ± 769.1	954 ± 242.3	154 ± 92.9	1171 ± 350.3	367 ± 98.8	131 ± 41.7	309 ± 99.2
Female age								
< 40	32	2212 ± 636.0	758 ± 193.8	61 ± 76.1	804 ± 295.5	241 ± 69.7	108 ± 29.5	240 ± 101.8
41–50	19	2256 ± 560.1	760 ± 150.2	75 ± 44.1	830 ± 275.3	259 ± 60.7	98 ± 27.0	233 ± 69.0
51–60	54	2135 ± 552.2	736 ± 178.1	78 ± 47.3	770 ± 262.9	243 ± 58.8	89 ± 25.3	219 ± 86.2
61–70	47	1926 ± 501.5	664 ± 155.8	87 ± 74.6	718 ± 233.5	201 ± 47.6	74 ± 17.6	182 ± 70.5
71–80	30	1860 ± 432.2	654 ± 109.6	96 ± 68.5	674 ± 239.9	195 ± 48.1	67 ± 15.4	174 ± 49.3
81–100	8	1579 ± 408.8	533 ± 125.5	132 ± 118.3	552 ± 158.4	173 ± 55.3	46 ± 9.8	142 ± 42.1
Male age								
< 40	9	3363 ± 889.0	1073 ± 246.8	143 ± 81.9	1189 ± 391.3	452 ± 116.2	170 ± 50.5	336 ± 96.9
41–50	25	3377 ± 759.1	1058 ± 246.6	183 ± 129.1	1211 ± 358.8	426 ± 89.5	156 ± 30.1	343 ± 115.7
51–60	39	3385 ± 819.3	1041 ± 247.5	149 ± 83.6	1311 ± 394.5	393 ± 98.4	149 ± 41.3	342 ± 104.6
61–70	41	2944 ± 603.0	908 ± 202.6	154 ± 86.5	1138 ± 299.2	334 ± 75.0	115 ± 30.3	295 ± 86.9
71–80	31	2726 ± 644.5	849 ± 198.2	138 ± 79.4	1038 ± 285.2	320 ± 76.7	108 ± 31.7	272 ± 75.6
81–100	9	2629 ± 536.7	754 ± 153.4	170 ± 73.7	1057 ± 255.1	311 ± 83.9	92 ± 19.0	245 ± 49.5

Abdominal muscle is defined as the portion of muscle that lies between the most inferior lung and the inferior aspect of L5.

### Muscle mass variation by sex and age

**Figure 6:**
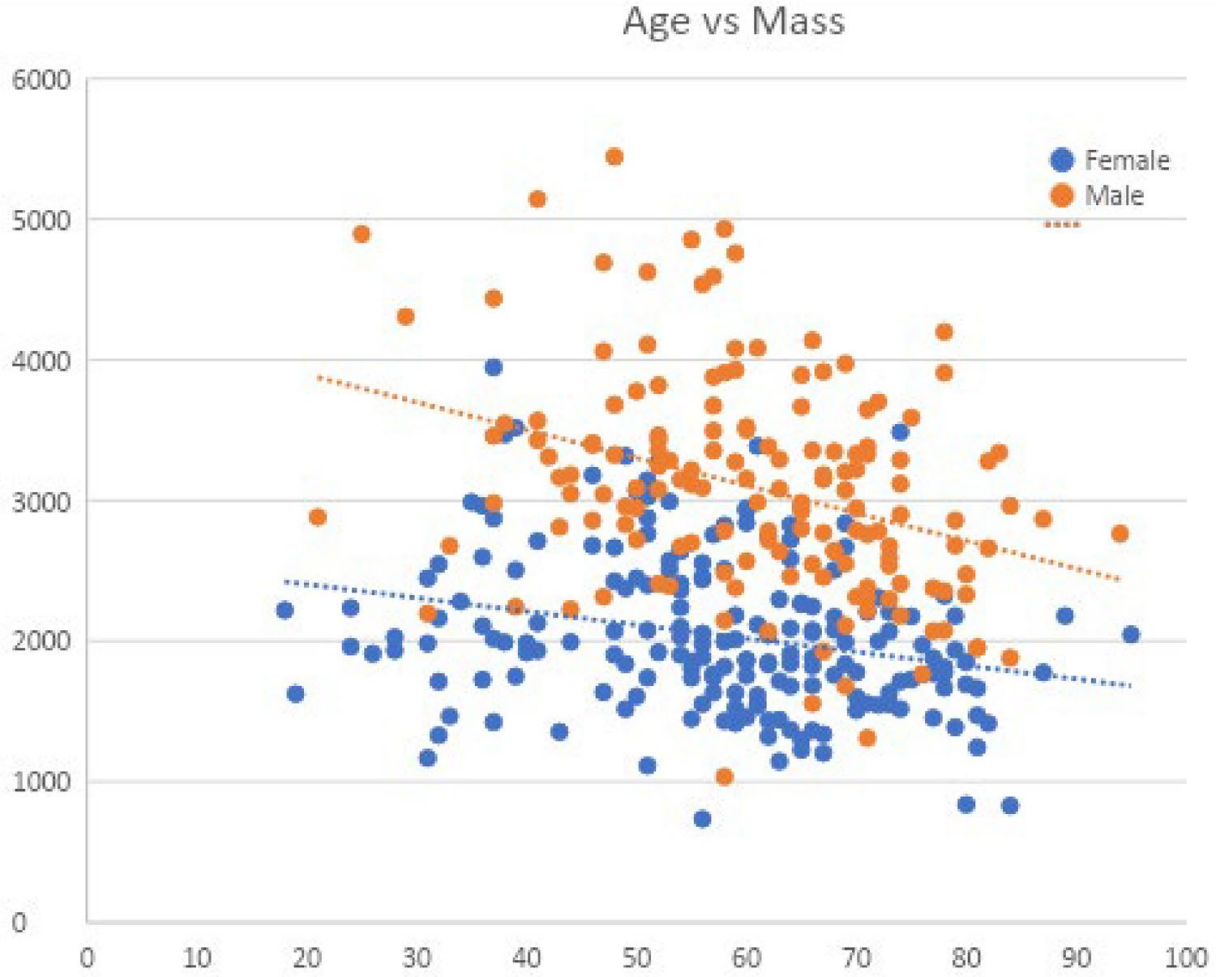
Scatterplot and slope of total muscle mass in grams (y-axis) plotted against age in years (x-axis). Correlation coefficients p-value < 0.0001.

We sought to determine the difference in total muscle mass between the sexes, defined as female and male, and determine the rate of decline in total muscle mass (measured in grams) by age (in years). For females, the average total muscle mass was 2041 ± 560.7 g with the highest average muscle mass recorded in the 41–50-year-old cohort at 2256 ± 560.1 g, with a change of − 0.96 g/year, declining to an average mass of 1579 ± 408.8 g in the 81–100-year-old cohort. For males, the average total muscle mass was 3086 ± 769.1 g with the highest average muscle mass measured in the 51–60-year-old cohort at 3385 ± 819.3 g, with a change of − 1.73 g/year declining to an average mass of 2629 ± 536.7 g in the 81–100-year-old cohort (Fig. 6). Total muscle mass and mass by muscle group by sex and age are listed in Table 3. p-values for correlation coefficients are < 0.0001.

The muscles most highly correlated with age are the bilateral quadratus lumborums for both sexes with a correlation coefficient of approximately − 0.5 for both males and females. Gluteus medius mass in females was positively correlated with age with a coefficient of 0.22. However, gluteus medius is also the only muscle group in men that has no negative correlation with age. Correlation coefficients for each muscle group are listed in Table 4, and scatterplots of individual muscle group variation are shown in Supplementary Fig. S4a.

**Table 4:** Sex-segregated correlation coefficients of muscle mass for different abdominal muscle groups with age.

Patient sex	Erector spinae	Gluteus medius	Lateral abdominals	Psoas	Quadratus lumborum	Rectus abdominis
Female	− 0.27 (p < 0.001)	0.22 (p = 0.002)	− 0.20 (p = 0.006)	− 0.33 (p < 0.001)	− 0.56 (p < 0.001)	− 0.30 (p < 0.001)
Male	− 0.39 (p < 0.001)	− 0.04 (p = 0.622)	− 0.19 (p = 0.017)	− 0.47 (p < 0.001)	− 0.55 (p < 0.001)	− 0.30 (p < 0.001)

When skeletal muscle mass (SMM) is adjusted by height and BMI respectively (Supplementary Table S3), the correlation coefficient with height decreased from 0.22 to 0.087, and the correlation with BMI decreased from 0.18 to − 0.059 (Supplementary Table S4). The correlation between SMM and both height and BMI was removed when BMI-adjusted z-scores of height-adjusted SMM were used (Supplementary Table S4). Previous findings regarding the correlation between age and muscle mass are maintained when muscle mass is adjusted by height and BMI (Supplementary Fig. S4b,c,e and Supplementary Table S1a,b,d). These trends are also maintained when the correlation analysis is applied to only patients over 40 years old (Supplementary Fig. S4d, Supplementary Table S1c).

## Discussion

We report a method to determine abdominal muscle mass from CT scans using deep learning and study mass variation by age and sex. The methods provide assessment of lean muscle mass for 12 distinct muscle groups from abdominal CT scans from clinically-indicated abdominal CT studies. The model was developed using data from participants of the Penn Medicine Biobank. When the model was deployed to a larger inferencing group, the results show the lean muscle mass by age and sex. There was a decline in muscle mass among all abdominal lean muscle groups with age. There was also a greater rate of decline of muscle mass in men than in women. These findings are consistent are consistent with our knowledge of age-related muscle loss in men and women^17^.

Many imaging tools employing different modalities and techniques exist for obtaining surrogate measures of muscle mass.^.^ While dual energy X-ray absorptiometry is a widely used tool for determining body composition, its use is limited by a lack of reference phantoms that precludes absolute calibration across manufacturers^30^. A systematic review conducted by Nijholt et al. examined the use of ultrasound for muscle mass assessment and found it to be a valid tool for measuring muscle size in adults, but highlighted its limitation in a clinical population due to altered tissue echogenicity^8^, in addition to being time-intensive and operator dependent. Praktiknjo et al. used lean muscle area measured on MRI as a marker for sarcopenia. Their study, however, measured only the erector spinae area at the level of the superior mesenteric artery, and it studied the association of erector spinae lean muscle area with post-transjugular intrahepatic portosystemic shunt procedure 1-year mortality and decompensation episodes^7^. Additionally, high costs and varying imaging protocols limit the generalizability of the MRI-based technique beyond the research setting^31^. Bioimpedance analysis (BIA) has been in use since the mid-1900s and is based in measuring tissue resistance to electrical conductivity to quantify body compartments. Its strengths are many, including low cost and portability, but it is limited by low reliability with high prediction error^32^ and a lack of standardized equations across patient populations^33^. In their meta-analysis, Amini et al. conclude that there is an unmet need for a reference standard for CT based tools for measuring muscle mass^34^.

Trends in CT utilization show a steady, general increase^35^, with over 70 million CT scans performed yearly in the US alone^36^. Mirroring this trend, CT-based tools for sarcopenia assessment are proliferating. At present, notable limitations to these efforts include variability in chosen muscle groups for measurement as imaging biomarkers, as well as inconsistencies in defining the different measured anatomical parts^34^. For instance, Burns et al. use a machine learning tool to assess for sarcopenia on abdominal CT at lumbar vertebral levels with high performance (DSC 0.896–0.916). Their aim is to generate automated measures of individual muscle cross-sectional areas at set lumbar vertebral levels to facilitate clinical use of historically established muscle-based imaging biomarkers^37^. Conversely, the algorithm presented in this paper functions to segment the volume of individual muscle groups spanning the entire abdomen, as defined in the methods, and derive a direct measurement of central muscle mass. Additionally, the aforementioned group selects for patients aged > 59 years-old and excludes cases with abdominal wall involvement or distortion by metastatic disease, organized collection, or defect. Other contemporaneous studies also utilize more homogenous patient samples, grouped by indication, such as asymptomatic patients presenting for a single scan type—a non-contrast enhanced screening colonography^16,17^. In contrast, our case set was selected at random (60% of cases) and enriched with scans containing pathology (40% of cases). This random selection of patients further advantages our study by allowing for a broad age range, and our case mix includes various scanning protocols, with and without intravenous contrast.

Upon literature review, a frequently encountered model for CT-based muscle segmentation is the use of a 2-step process which first delineates an area of interest by automatic identification of specific vertebral endplate levels and then segments muscle within that area. Across recent publications, two neural networks are routinely used to this end, and they are the automated spinal column extraction network by Yao et al.^38^ which was first used on a large patient cohort by Burns et al.^37^, and the automated muscle segmentation network designed by Ronneberger et al.^23^. These networks are used by Graffy et al.^17^, Bridge et al.^15^, and Pickhard et al.^16^ in the same manner. In these studies, the output is a measurement of cross-sectional muscle area and attenuation at different vertebral body levels. The cross-sectional area is converted to a volumetric measure by Graffy et al. using a formula of pixel area × craniocaudal slab thickness applied only at the L3 level for analysis. Our novel technique also uses a two-step approach, but instead begins with delineating the boundaries of the abdominal cavity, followed by semantic segmentation and direct measurement of whole muscle volume using an original U-Net, as opposed to segmentation of muscle cross-sectional area at set vertebral body levels. This minimizes the error introduced by patient position and posture on the gantry, or by spinal variations or deformities when they occur.

Furthermore, our aim was to develop a fully automated deep learning method for large-scale generation of muscle IDPs from clinical CT scans. Though deep learning methods have been developed to this end as detailed above^34^, the integration of multiple algorithms for abdominal compartment identification and the determination of abdominal muscle from 12 distinct muscle groups using multiclass semantic segmentation has not been performed and is essential to deploy fully automatic methods in routine clinical application. In addition to providing a direct and incontrovertible measure of central muscle mass, this technique provides granular assessment of individual muscle groups, which has not before been studied at scale.

The quadratus lumborum was the muscle group most highly correlated with aging. Comparison to results in existing literature is limited by the rare use of the direct measurement of muscle mass in grams as a descriptor, and by our cross-sectional study design which captures patients of a wide age range and permits us to report on muscle mass variation by age. The most commonly reported measurements in the literature are otherwise muscle attenuation and cross-sectional area at specific vertebral body levels^16^ and cross sectional area with derivation of the skeletal muscle index^37^. One study reports a mean total abdominal muscle volume of 63 ± 8 cm^3^ measured on a 5 mm abdominal CT slice at the L4-L5 level in an all-male cohort without female subjects or commentary on variation^39^. Graffy et al. report a change in muscle cross sectional area per year measuring − 2.16 cm^2^ for males and − 0.91 cm^2^ for females^17^. In a 2012 literature review by Mitchell et al., it is noted that the median percentage value for rate of muscle loss is 0.64–0.7%/year in women and 0.8–0.98%/year in men^40^. These study results are in keeping with our observation of a slower rate of decline in women. An MRI-based protocol measuring whole body skeletal muscle volume, executed on a cohort of adult men and women, also revealed a negative correlation between whole body skeletal muscle mass and age. Regression analysis resulted in a slope of − 0.19 in men and − 0.08 in women when plotting relative skeletal muscle mass (body mass/skeletal muscle mass) against age^41^. While direct comparison is not possible because our metric unit is different, these findings mirror our obtained coefficients of a − 17.3 g/year change in men and − 9.6 g/year change in women.

We encountered limitations to this study. We measured abdominal muscle mass on CT using deep learning algorithms for multiclass semantic segmentation. This algorithm produces error rates that depend on the muscle group. In particular, error rates for the erector spinae and gluteus medius were the lowest (< 10% Dice score error), followed by the rectus abdominis, psoas and lateral abdominal muscles (< 15% error). The quadratus lumborum had the lowest performance with an error rate approaching 25%. This is most likely related to difficulties in delineating the quadratus lumborum from the underlying psoas muscle in the absence of a clear fat plane. While the lateral abdominal muscle error rates were low (< 15% error), an audit of the segmentations revealed sporadic inclusion of the inferior tip of the latissimus dorsi within the labeled “lateral abdominal” outline, which overestimates the latter’s true contribution to central muscle mass. The variability in intraclass correlation coefficients among different muscle groups appears to depend mainly on the overall difficulty of identifying the muscle group (i.e. generation of the ground truth label by the expert). Due to indistinctness of lateral muscle groups by computed tomography, we combined the lateral abdominal wall muscles consisting of the external and internal obliques and transversus abdominis into a “lateral abdominals” muscle group. We obtained muscle mass from volume by a simple conversion, using a muscle density of 1.06 kg/L, which introduces a small margin of error due to fatty atrophy. Based on fat and muscle densities of 0.9 g/cm^3^ and 1.06 g/cm^3^ respectively, muscle is only 17% denser than fat. Taking an extreme example of 50% muscle replacement by fat, this would introduce only an 8% error, which may have been large enough in this small sample to distort the results of older age cohorts who tend to experience greater fatty atrophy than younger groups. Additionally, this algorithm was applied to patients in a single large hospital system and thus our findings could be influenced by Berkson’s bias and skewed towards individuals with specific health conditions. The population of this study is overall comparable in age and sex to the Penn Medicine Biobank as a whole (Supplementary Table S2)^45^. The selection bias along with our limited sample size and data variability currently limits the generalizability of our findings, which are preliminary and mainly exploratory. However, our study helps to establish a proof-of-concept for using deep learning to determine image-derived phenotypes and their association with risk factors for sarcopenia and can serve as a foundation for future research with larger sample sizes and more specific hypotheses.

We determined abdominal muscle mass in a large cohort of medical biobank patients and obtained preliminary information about the variations in this IDP by age and sex. The methodology obtains highly accurate segmentations of 6 distinct abdominal muscle groups on each side of the body. A key advantage of our institutional biobank is that it allows for the measurement of the mass of individual muscle groups on thousands of patients; moreover, the presence of rich clinical data allows stratification of muscle mass across various clinical domains—age, gender, ethnicity, disease type, etc. With the current aging trends, the number of sexagenarians and older is projected to double by the year 2050^42,43^. This trend will see a commensurate increase in rates of chronic illness and secondary sarcopenia, as well as primary (age-related) sarcopenia. In a precision medicine approach, this objective, quantitative reference standard will have a key role in differentiating “normal” or primary sarcopenia from accelerated muscle loss due to genetic risk factors or illness, allowing early intervention where possible, which will be key to alleviating the associated public health burden which stands to be substantial at this scale^44^.

Future work should replicate and corroborate these findings at other institutions with larger sample sizes and further refine the algorithm by presenting it with additional diverse pathologies. The use of imaging phenotypes will enable investigation of how variations in individual muscle mass affect surgical outcomes. Future studies should investigate the genetic loci associated with either high or low muscle mass. This could help to identify additional genetic loci/pathways that could be future targets for therapy. This is a unique and key strength of the resource, making it a sure step towards transforming the diagnostic paradigm of sarcopenia and advancing the field of sarcopenia research and management.

### Supplementary Information

Supplementary Information.

## Data Availability

The data that support the findings of this study can be made available under controlled access to protect patient privacy. There may be restrictions on data use as defined in the data usage agreement. Responses to requests for access to data will be handled within 10 business days of the request. Please contact W.R.W. to discuss data access.
