# Supplementary material for: CT imaging-derived phenotypes for abdominal muscle and their association with age and sex in a medical biobank
Source: Sci Rep. 2024 Jun 26;14:14807. doi: 10.1038/s41598-024-64603-6 (PMC11208425; doi:10.1038/s41598-024-64603-6)
Supplement: Supplementary file 1 — Supplementary Information. [file 41598_2024_64603_MOESM1_ESM.docx]

**Supplementary Information**

**Supplementary Figures**


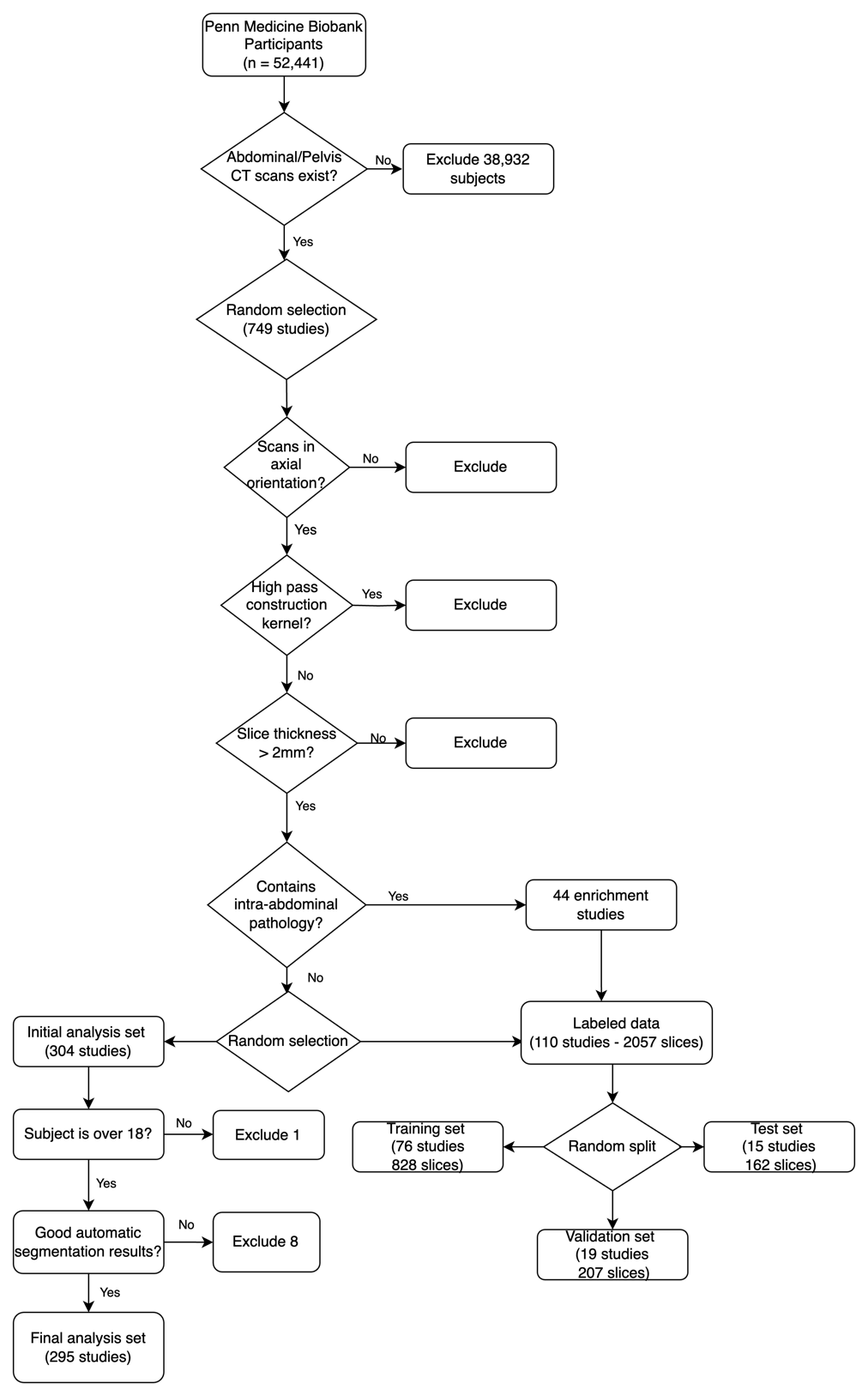


**Figure S1.** Flowchart of cases with inclusion and exclusion criteria. The enrichment dataset included diverticulitis, sarcopenia, colon cancer, umbilical hernia, cholelithiasis, cholecystitis, ulcerative colitis, intestinal fistula, abdominal abscess, small bowel obstruction, pyelonephritis, Crohn’s disease, diverticulitis with hemorrhage, hepatomegaly, splenomegaly, short bowel syndrome, kidney stone, ventral hernia, and perianal fistula


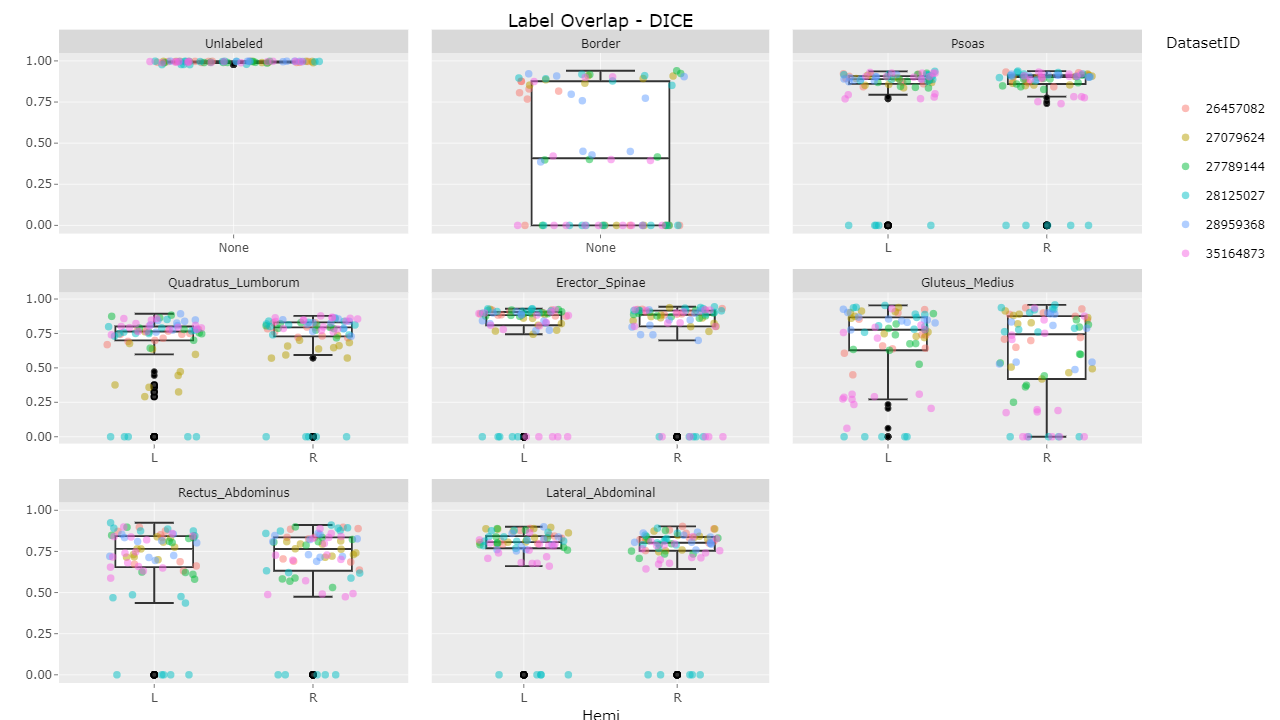


**Figure S2.** Distribution of Dice scores for different muscle groups in 6 studies labeled by all annotators to determine inter-rater variability. Each study is labeled by a DatasetID with a different colored circle, and same-colored circles represent different annotators of the same study.


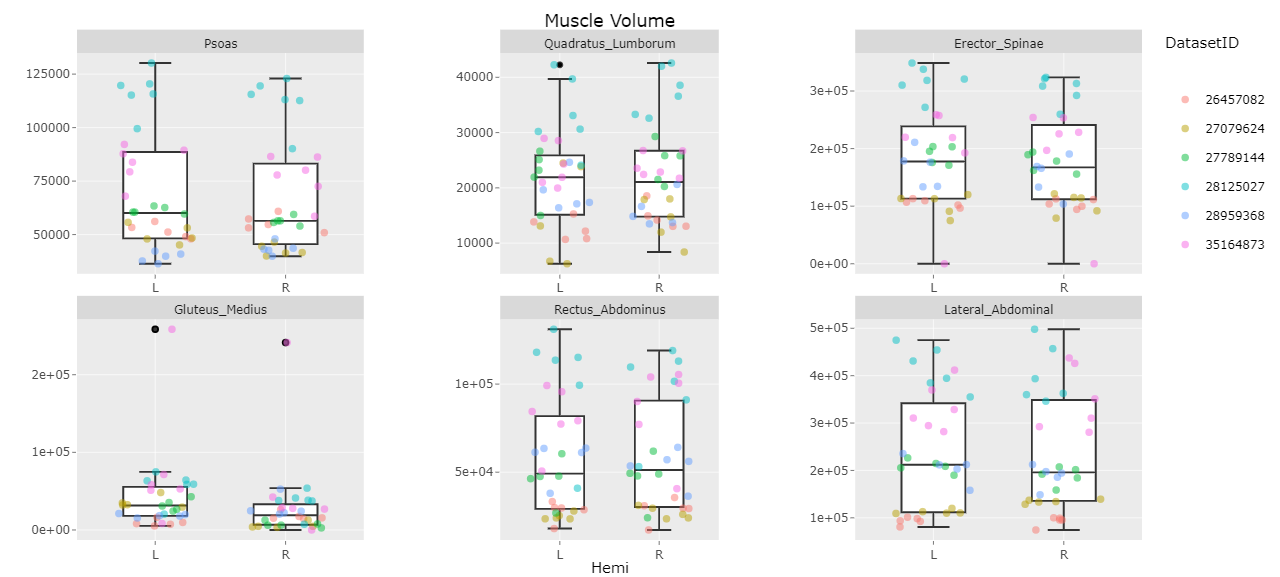


**Figure S3.** Distribution of muscle volume (in pixel volume units) for different muscle groups in 6 studies labeled by all annotators to determine inter-rater variability. Each study is labeled by a DatasetID with a different colored circle, and same-colored circles represent different annotators of the same study.


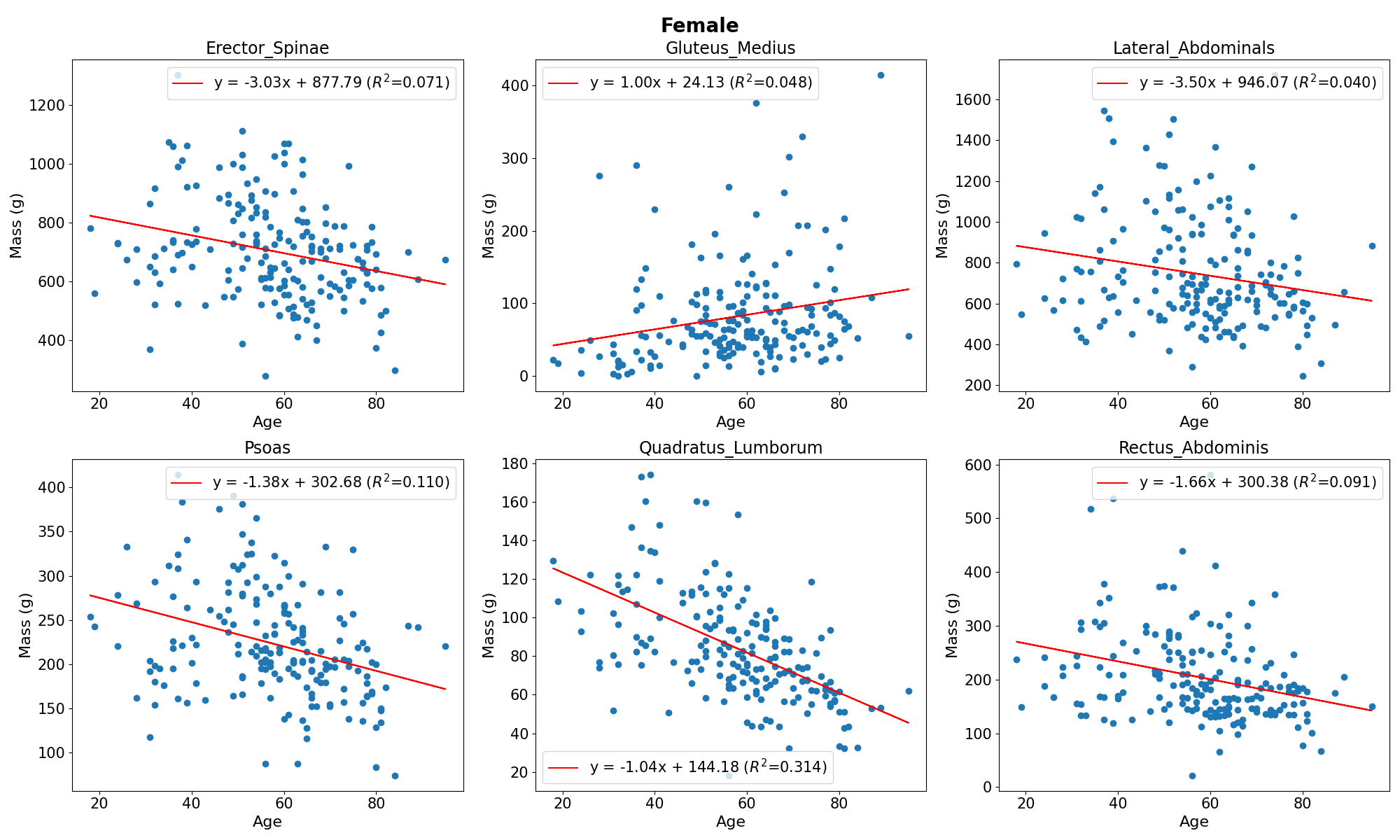


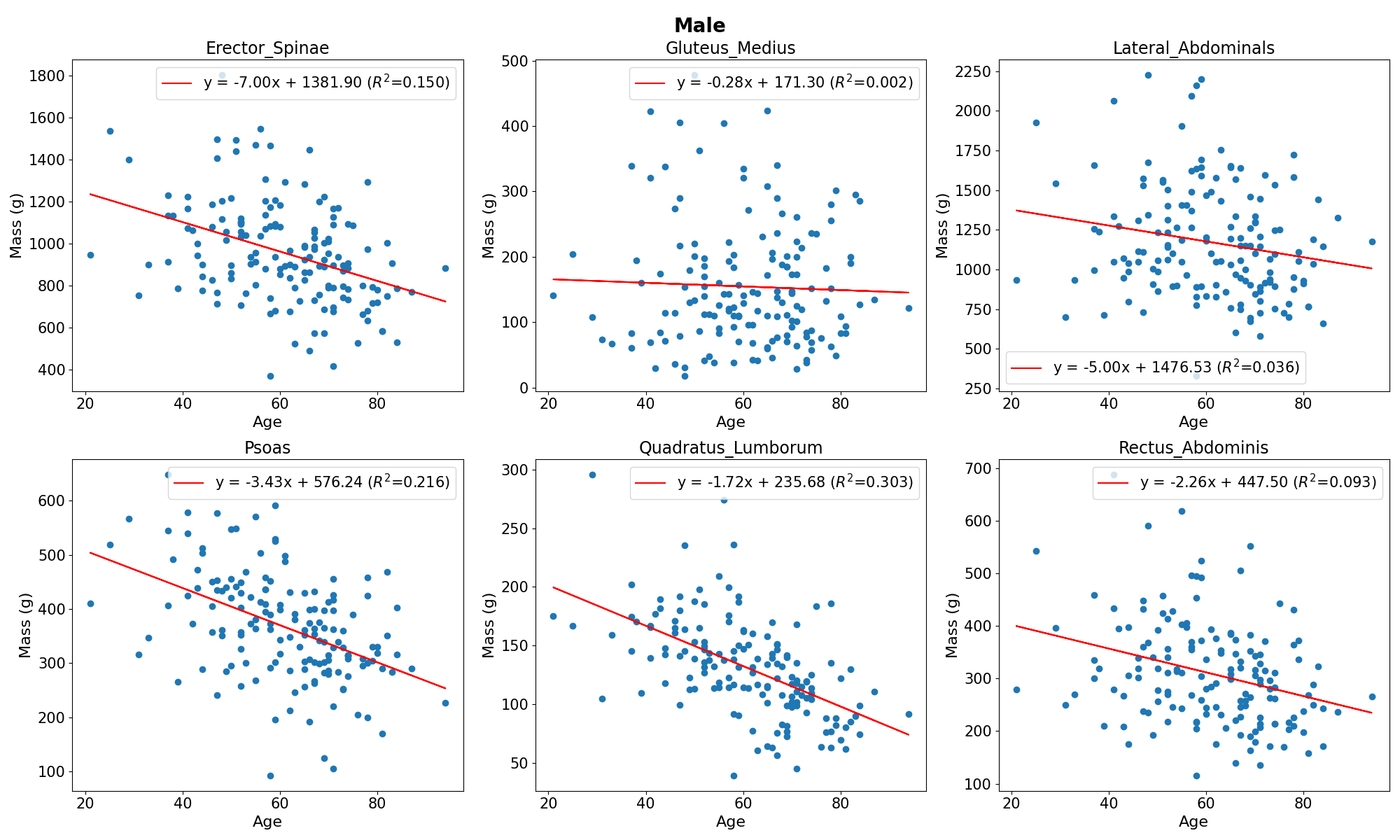


**Figure S4a.** Scatterplots of muscle mass in grams of individual muscle groups (y-axis) plotted against age in years (x-axis) in male and female subjects.


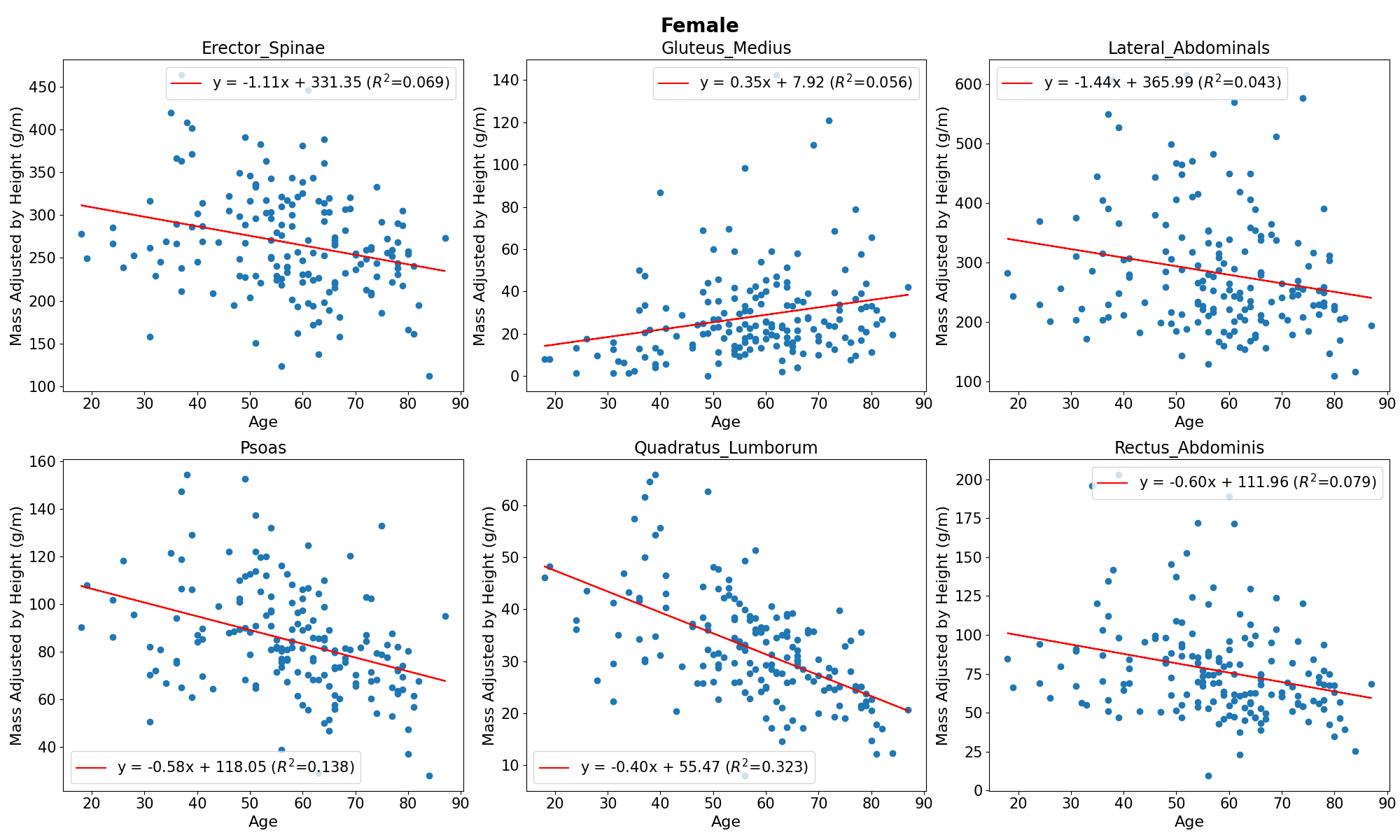

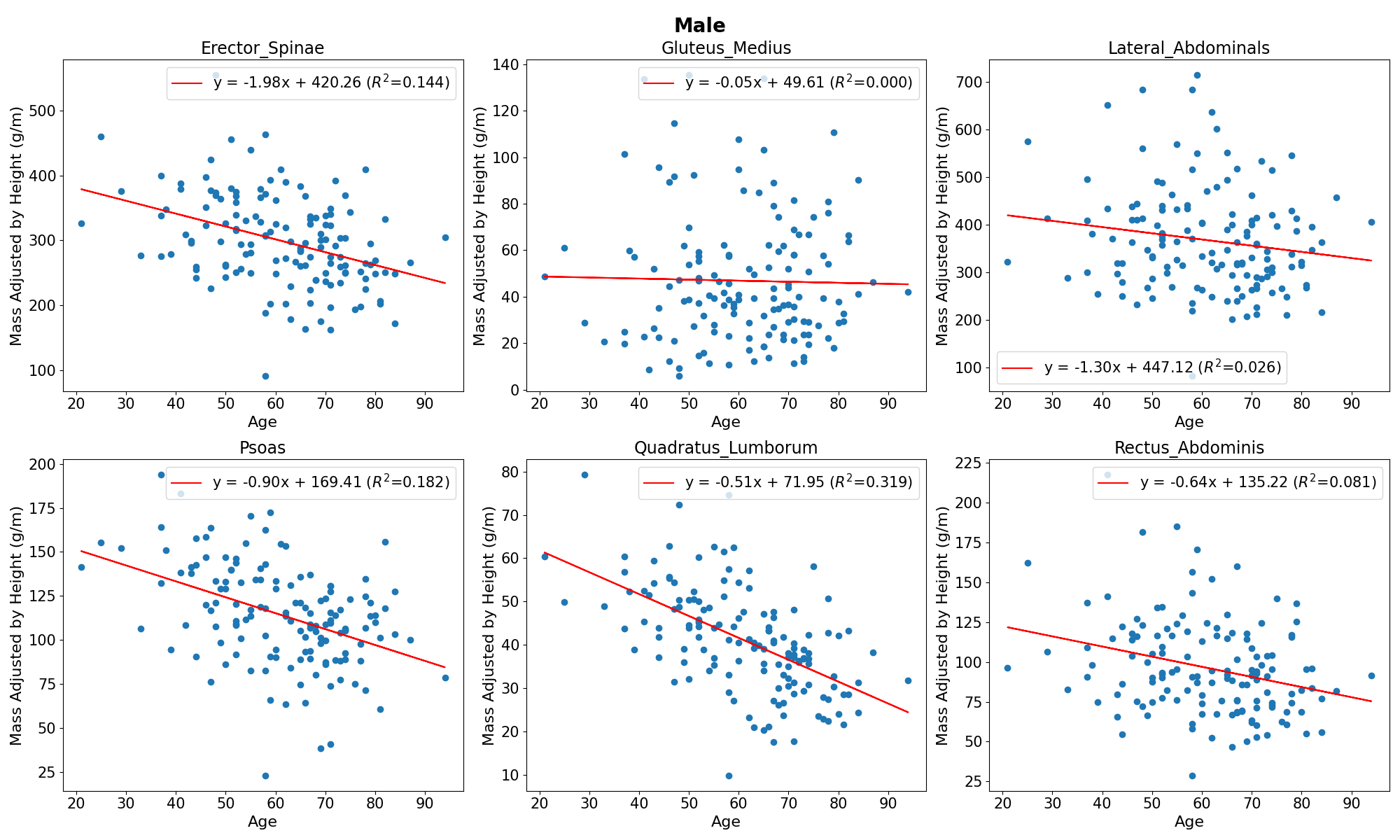


**Figure S4b.** Scatterplots of muscle mass **adjusted by height** of individual muscle groups (y-axis) plotted against age in years (x-axis) in male and female subjects.


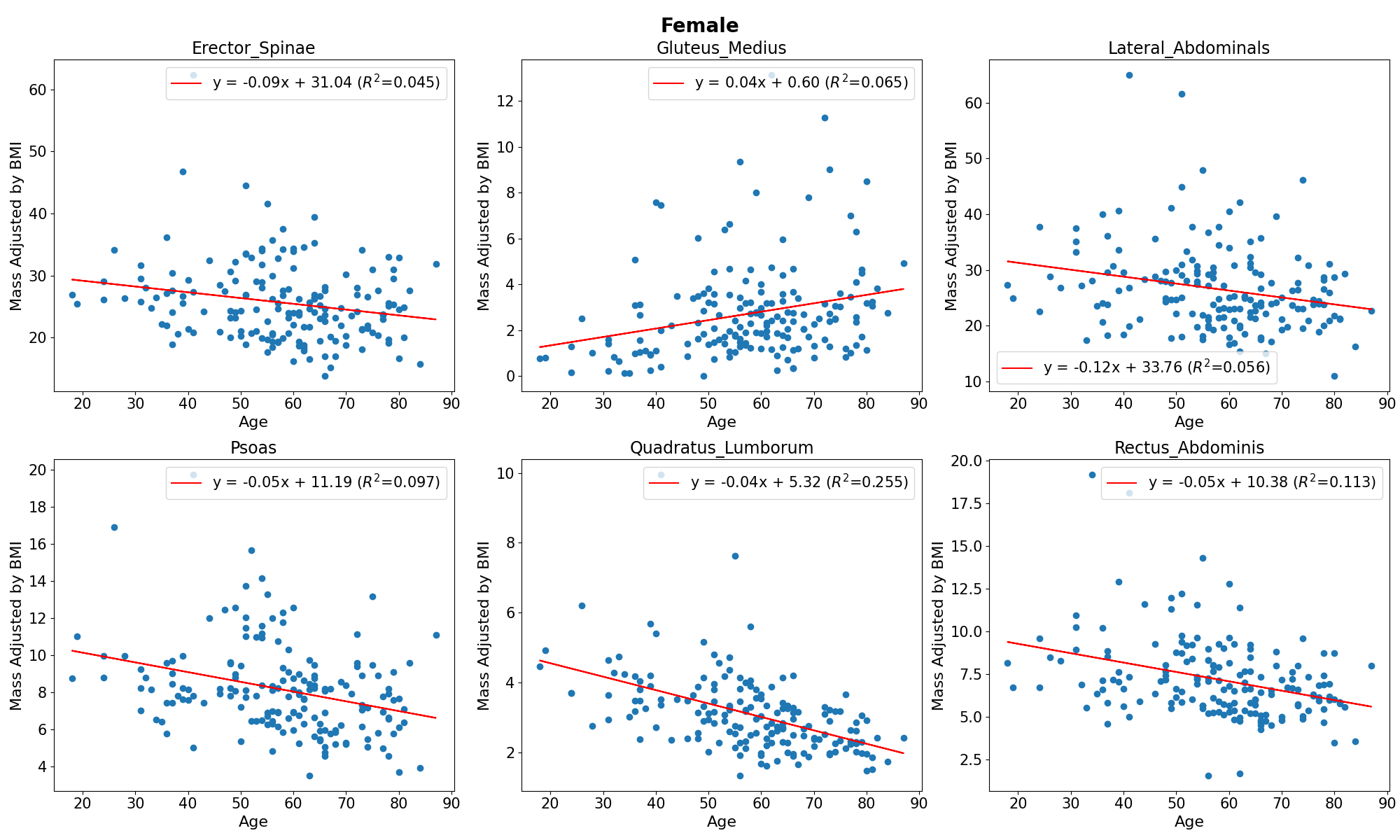

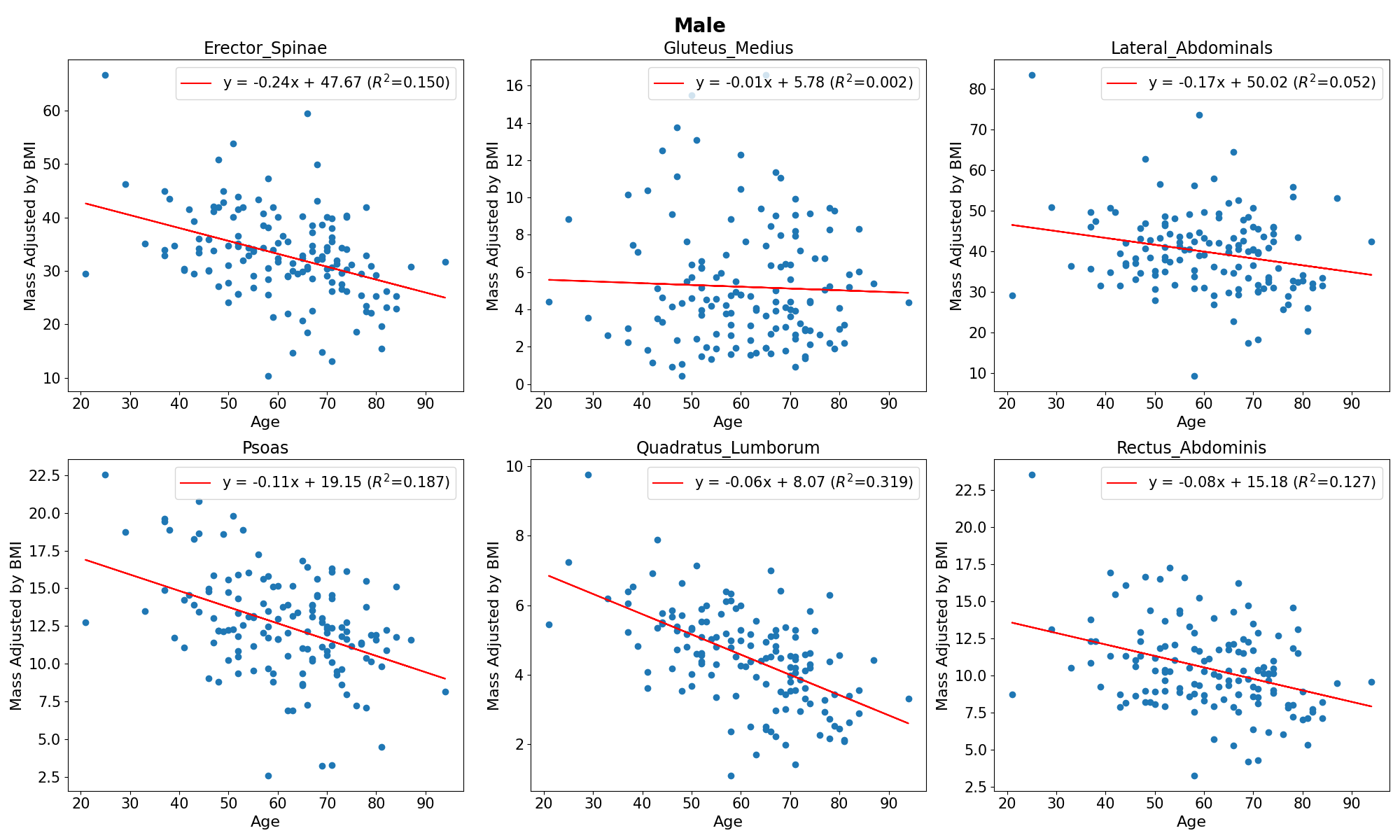


**Figure S4c.** Scatterplots of muscle mass **adjusted by BMI** of individual muscle groups (y-axis) plotted against age in years (x-axis) in male and female subjects.

**
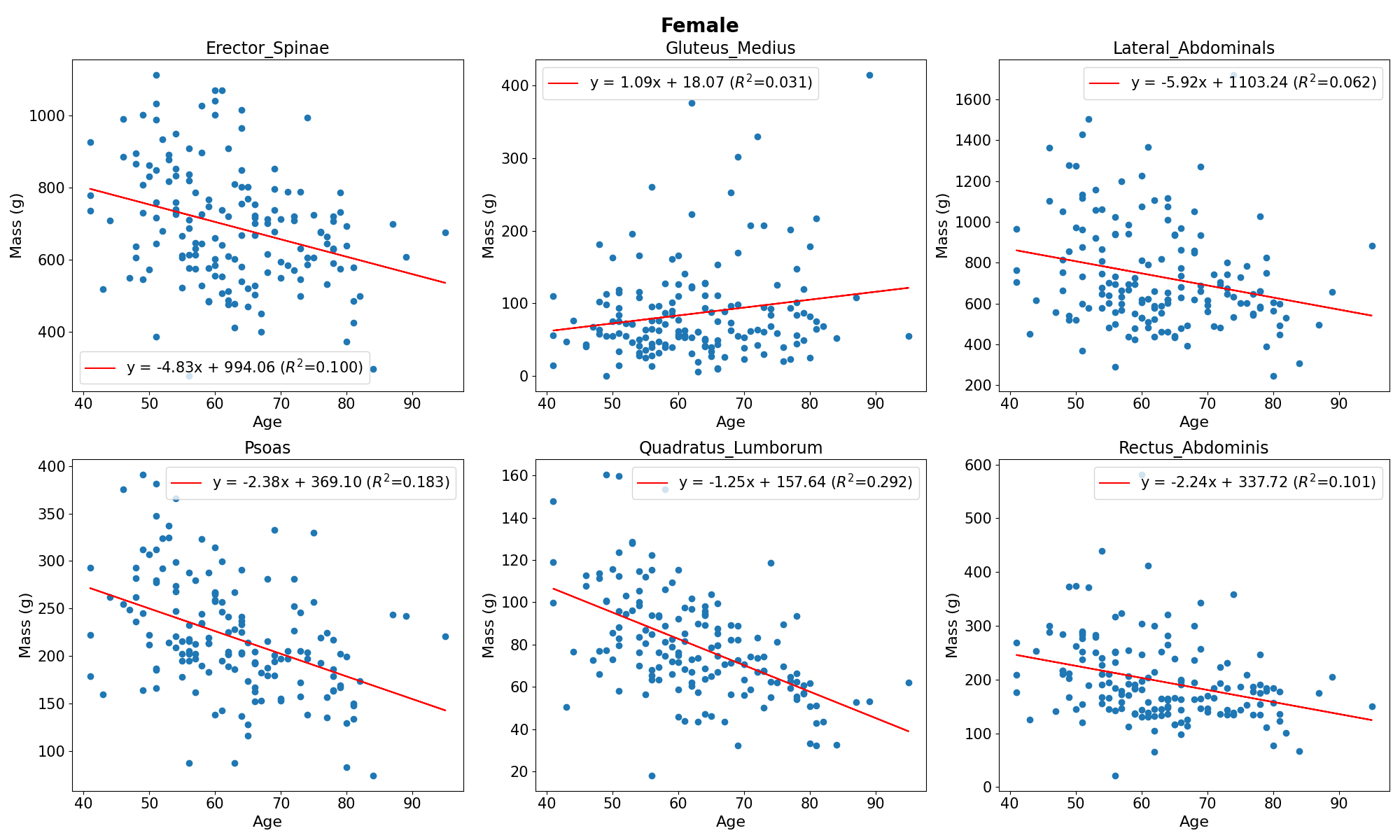

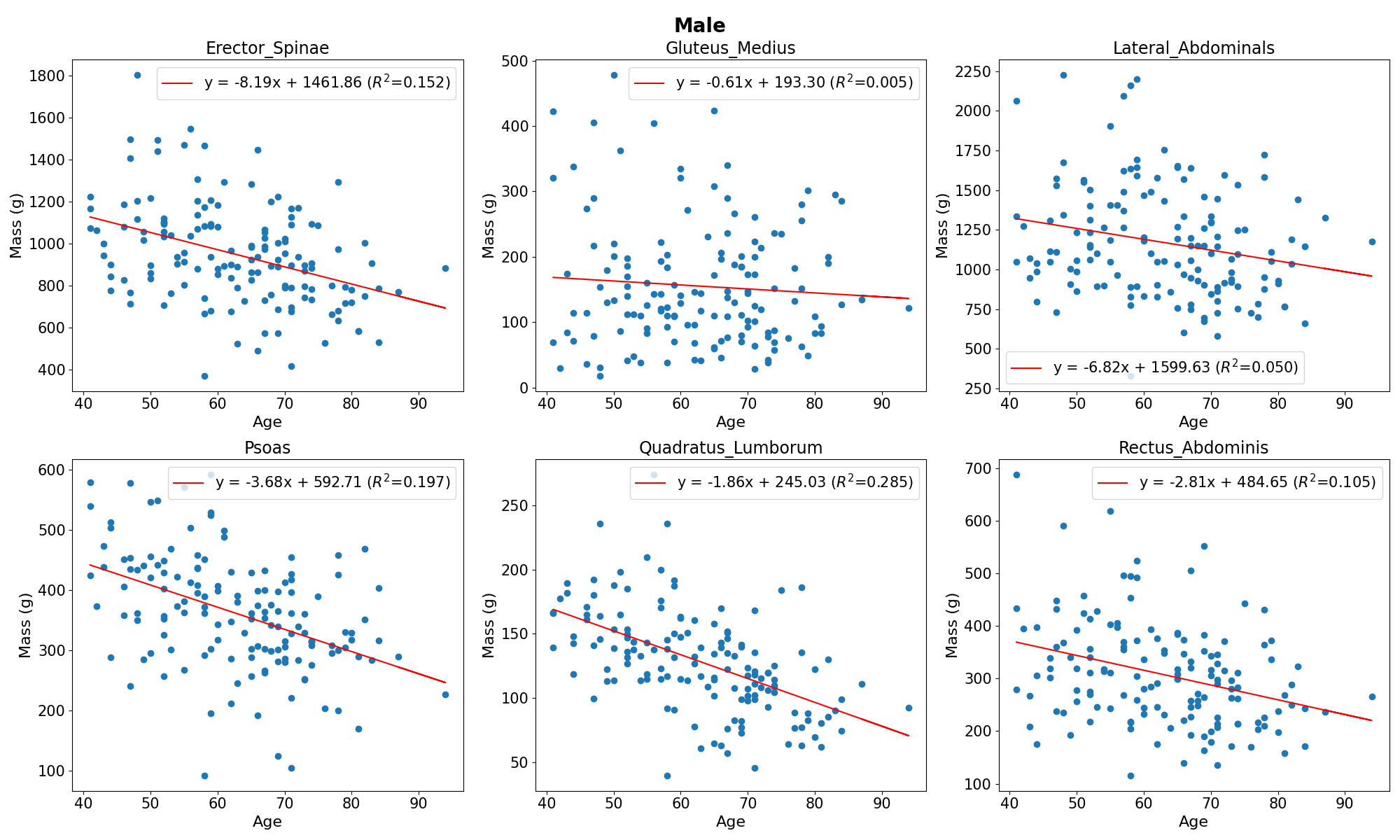
**

**Figure S4d.** Scatterplots of muscle mass in grams of individual muscle groups (y-axis) plotted against age in years (x-axis) in male and female subjects **over 40 years old**.


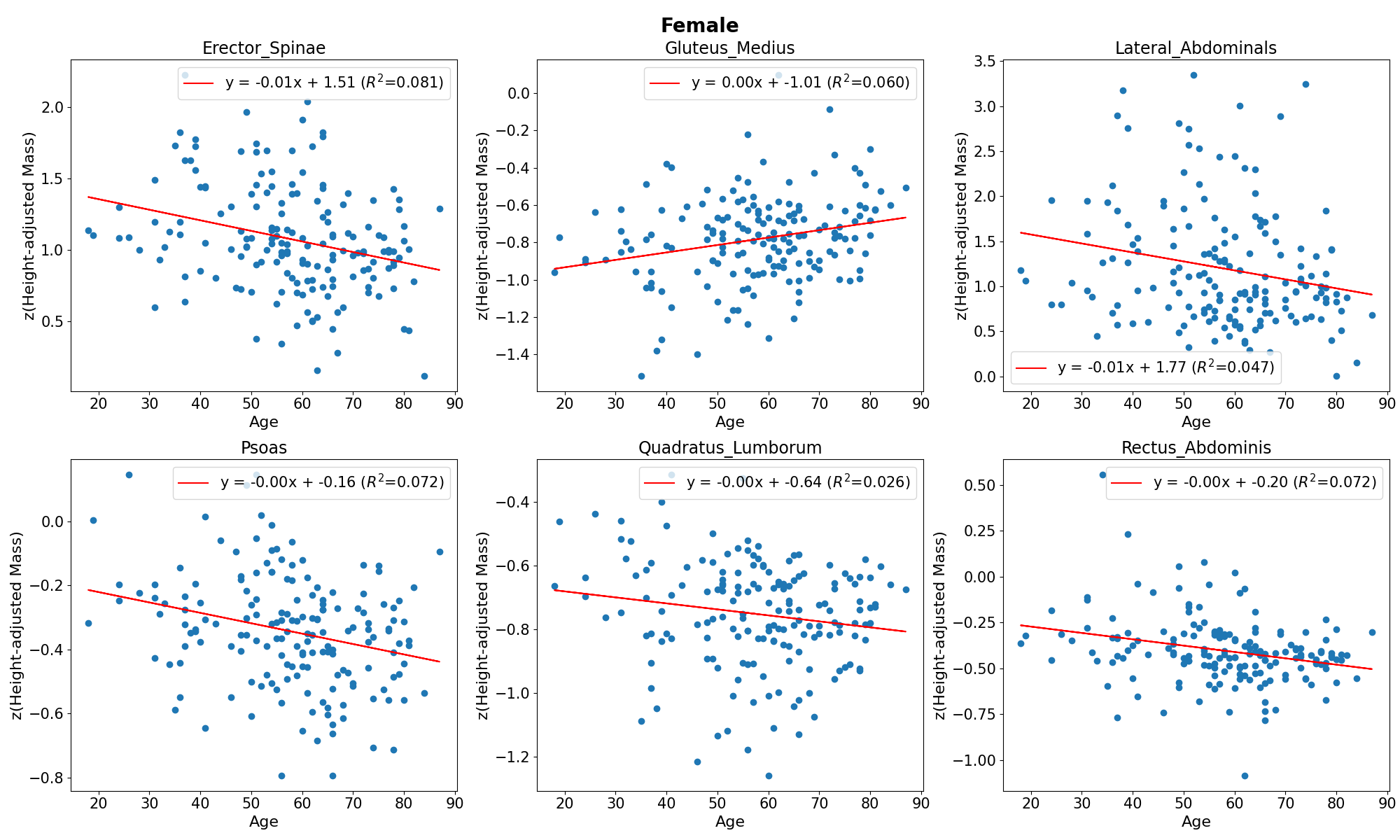

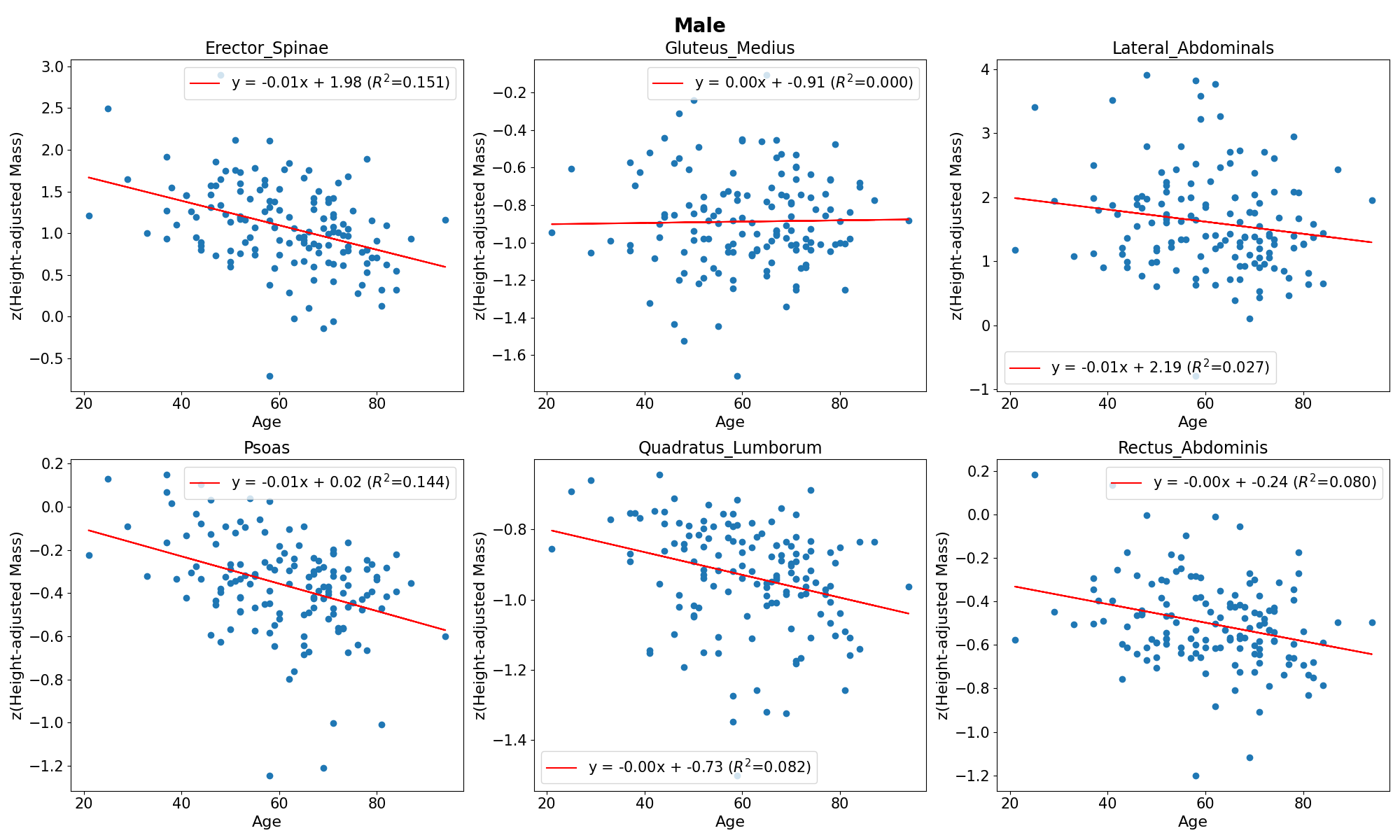


**Figure S4e.** Scatterplots of **BMI-adjusted** **z-scores of height-adjusted muscle mass** for individual muscle groups (y-axis) plotted against age in years (x-axis) in male and female subjects.

**Table S1a.** Sex-segregated correlation coefficients of muscle mass adjusted by height for different abdominal muscle groups with age.

| **Patient Sex** | **Erector Spinae** | **Gluteus Medius** | **Lateral Abdominals** | **Psoas** | **Quadratus Lumborum** | **Rectus Abdominis** |
| --- | --- | --- | --- | --- | --- | --- |
| **Female** | -0.26 (p<.001) | 0.24 (p=0.002) | -0.21  (p=0.007) | -0.37 (p<.001) | -0.57  (p<.001) | -0.28  (p<.001) |
| **Male** | -0.38 (p<.001) | -0.02 (p=0.793) | -0.16  (p=0.057) | -0.43 (p<.001) | -0.56  (p<.001) | -0.29  (p<.001) |

**Table S1b.** Sex-segregated correlation coefficients of muscle mass adjusted by BMI for different abdominal muscle groups with age.

| **Patient Sex** | **Erector Spinae** | **Gluteus Medius** | **Lateral Abdominals** | **Psoas** | **Quadratus Lumborum** | **Rectus Abdominis** |
| --- | --- | --- | --- | --- | --- | --- |
| **Female** | -0.21 (p=0.006) | 0.26  (p<.001) | -0.24  (p=0.002) | -0.31 (p<.001) | -0.51  (p<.001) | -0.34  (p<.001) |
| **Male** | -0.39 (p<.001) | -0.04 (p=0.634) | -0.23  (p=0.006) | -0.43 (p<.001) | -0.56  (p<.001) | -0.36  (p<.001) |

**Table S1c.** Sex-segregated correlation coefficients of muscle mass adjusted by height for different abdominal muscle groups with age **for patients over 40 years old**.

| **Patient Sex** | **Erector Spinae** | **Gluteus**  **Medius** | **Lateral Abdominals** | **Psoas** | **Quadratus**  **Lumborum** | **Rectus Abdominis** |
| --- | --- | --- | --- | --- | --- | --- |
| **Female** | -0.32  (p<.001) | 0.18 (p=0.027) | -0.25  (p=0.002) | -0.43 (p<.001) | -0.54  (p<.001) | -0.32 (p<.001) |
| **Male** | -0.39  (p<.001) | -0.07 (p=0.376) | -0.22  (p=0.007) | -0.44 (p<.001) | -0.53  (p<.001) | -0.32 (p<.001) |

**Table S1d.** Sex-segregated correlation coefficients of BMI-adjusted z-scores of height-adjusted muscle mass for different abdominal muscle groups with age.

| **Patient Sex** | **Erector Spinae** | **Gluteus**  **Medius** | **Lateral Abdominals** | **Psoas** | **Quadratus**  **Lumborum** | **Rectus Abdominis** |
| --- | --- | --- | --- | --- | --- | --- |
| **Female** | -0.28  (p<.001) | 0.25  (p=0.001) | -0.22  (p=0.005) | -0.27 (p<.001) | -0.16  (p=0.035) | -0.27 (p<.001) |
| **Male** | -0.39  (p<.001) | -0.02 (p=0.828) | -0.16  (p=0.052) | -0.38 (p<.001) | -0.29  (p<.001) | -0.28 (p<.001) |

**Table S2.** Demographics of PMBB Participants and Participants in this Study

|  | **PMBB Participants**  **(n = 174712)** | **Participants in this Study**  **(n = 346)** |
| --- | --- | --- |
| **Gender (%)** |  |  |
| Male | 44.1 | 45.1 |
| Female | 55.9 | 54.9 |
| **Age groups (%)** |  |  |
| (0, 40] | 25.9 | 12.5 |
| (40, 50] | 14.8 | 12.7 |
| (50, 60] | 19.4 | 26.8 |
| (60, 70] | 23 | 25.4 |
| (70, 80] | 16.4 | 17.6 |
| (80, 120] | 5 | 4.9 |

**Table S3**. Association between the z-score for height-adjusted muscle mass (SMM) and covariates using the model proposed in Derstine, et al. “Optimal body size adjustment of L3 CT skeletal muscle area for sarcopenia assessment.” (2021)

$SMM=\beta_{0}+\beta_{1} x BMI+\beta_{2} x sex+\beta_{3} x sex x BMI$.

| beta | model value | Covariates | p-value |
| --- | --- | --- | --- |
| $\beta_{0}$ | 33.0 |  |  |
| $\beta_{1}$ | 3.38 | BMI | 2.85e-192 |
| $\beta_{2}$ | 25.1 | sex | 1.26e-2 |
| $\beta_{3}$ | 0.11 | sex x BMI | 2.17e-1 |

**Table S4.** Pearson Correlation Coefficients between SMM (skeletal muscle mass) related values and Height/BMI

| Value | Height | BMI |
| --- | --- | --- |
| SMM | 0.22  (p < .001) | 0.18  (p <.001) |
| Height-adjusted SMM | 0.087  (p < .001) | N/A |
| BMI-adjusted SMM | N/A | -0.059  (p = 0.01) |
| BMI-adjusted z-scores of height-adjusted SMM | 8.6e-05  (p = 0.997) | -1.08e-15  (p = 0.999) |
